# Identification of Preferred Learning Style of Medical and Dental Students Using VARK Questionnaire

**DOI:** 10.1155/2021/4355158

**Published:** 2021-10-18

**Authors:** Ayesha Fahim, Saba Rehman, Fariha Fayyaz, Mariyah Javed, Muhammad Anwaar Alam, Sadia Rana, Fahim Haider Jafari, Mohammad Khursheed Alam

**Affiliations:** ^1^University College of Dentistry, University of Lahore, Lahore, Pakistan; ^2^Azra Naheed Dental College, Lahore, Pakistan; ^3^Sharif Medical and Dental College, Lahore, Pakistan; ^4^Sahara Medical College, Narowal, Sahara, Pakistan; ^5^College of Dentistry, Jouf University, Sakaka, 72345 Al-Jouf, Saudi Arabia

## Abstract

Students have unique preferences when it comes to knowledge acquisition, information processing, retention in memory, and recall. This study is aimed at examining the preferred learning styles of medical and dental undergraduate students of Pakistan. It is also aimed at investigating the influence of gender, preclinical or clinical academic year, and academic record on the preferred learning style. A descriptive cross-sectional study was conducted in Pakistan. The learning styles of undergraduate students were identified using visual, aural, read/write, and kinesthetic (VARK) questionnaire. Students were also asked about their satisfaction towards teaching style of their teachers in institute. Descriptive statistics were done to characterize the learning styles of the students. The Fisher test and chi-square test were used to compare the learning preferences between genders and public/private sector students and among preclinical/clinical years. A *p* value of less than 0.05 was considered significant. A total of 1473 students participated in the study. Among the students, 39.37% preferred unimodal learning style whereas 60.62% preferred multimodal style. Kinesthetic (K) and visual (V) were the most preferred unimodal styles. The preferred learning styles of female students are aural (A), visual (V), and kinesthetic (K), whereas male students preferred visual (V) and kinesthetic more (K). Students with lower academic record chose unimodal styles in comparison to high achievers that chose multimodal styles. Students of clinical year preferred multimodal and quadmodal styles in comparison to preclinical year students. An alarming 78% of students were dissatisfied with their teacher's instructional style. Majority of students prefer multimodal learning styles over unimodal style. Gender, public/private sector, and academic record have influence on the preference of learning styles. Majority of the students are dissatisfied with their teacher's instructional style and rely on social media platforms for understanding. Academics need to adapt their teaching methods according to student preferences in order to get better graduates.

## 1. Introduction

Education is the process of facilitating learning, or the acquisition of knowledge, skills, values, beliefs, and habits [1]. The manner in which a student prefers to acquire, process, memorize, and recall information is described as the individuals' learning style [2]. Each individual has his own unique style of learning. A single student may adapt single or multiple modes of acquiring knowledge [3]. Academics view learning style in terms of factors that affect the learning of students due to individual preferences for physical, social, and the environmental elements in the course of learning [4].

For the past few decades, medical education has been rapidly shifting from teacher-centered passive to student-centered active approach. Thus, awareness about preferred learning style can be useful for both pupils and educators [5]. Instructors can modify their teaching according to learner's preference by adapting multiple teaching modalities. Student motivation and performance improve when instruction is molded to student learning styles [6]. Similarly, knowing one's learning style can minimize learning time, enhance student engagement in the process, and increase his learning outcome and efficiency [7].

Different models have been developed over time to indicate students' overall approaches to learning and their perceptions of the teaching-learning environments. Popular models include Riechman and Grasha learning style [8], Dunn and Dunn learning style [9], Kolb's learning style [10, 11], Gregorc learning style [11], Reid's learning style [12], and McCarthy learning style [13].

Various studies have been conducted around the world in different departments to identify learning preference [14]. One of the most commonly used models is the VARK model designed by Fleming and Baume, which categorizes learning preferences into four modes of sensory pathways: visual (V), aural (A), read/write (R), and kinesthetic (K) [15]. These different learning styles were acknowledged after thousands of hours of classroom observation [16]. Every student has his own preference to a particular approach [17].

In the past two years, medical education in Pakistan has rapidly shifted to online and blended (part online, part on-campus) education. In the recent past, even with technological advancement, students mainly relied on textbooks for gaining new knowledge in the traditional education system [18]. With the advent of online education system, teachers have to modify the teaching learning to adapt to student's increasing educational demands [19]. Thus, to make an effective educational curriculum, it is important to identify students' learning style preferences. In the field of healthcare sciences, teaching is a combination of cognitive and practical elements, which enhances student participation utilizing various sensory inputs to equip students with the knowledge and practice of patient handling. Single teaching modality is never enough. Thus, it is imperative that educators identify preferred learning styles of undergraduate medical and dental students.

This study is aimed at examining the preferred learning styles of medical and dental undergraduate students of Pakistan. It is also aimed at investigating the influence of gender, academic year, and preclinical or clinical and academic record on the preferred learning style.

## 2. Methods

### 2.1. Study Design

A descriptive cross-sectional study was done from April to June 2021, to determine learning preferences of students of medical and dental colleges of Pakistan. The study was undertaken according to the principles of ethics of Declaration of Helsinki, and ethical approval was obtained from institutional review board (ANDC/RAC/34/06) of Azra Naheed Dental College, Lahore, Pakistan.

### 2.2. Instrument

To determine whether a particular teaching method might enhance student's learning process, a survey was conducted in Pakistan using anonymous online questionnaire. The questionnaire consisted of three parts:

1. Initial introduction and objectives of the study were explained followed by the statement of consent. Participant information sheet was provided which stated that student participation is purely voluntary, and it will not affect their assessment or performance in anyway. Student's demographics include age, gender, specialty of study MBBS/BDS, degree program (public/private), year of study, and obtained percentage in the last professional exam (%). Years 1, 2, and 3 of MBBS and years 1 and 2 of BDS were considered preclinical years, and the last two professional years were considered clinical years. Students' names and names of institutions were not asked to maintain anonymity of research and maintain participant confidentiality
2. The 14 questions of VARK questionnaire version 8.01. “VARK” is used to describe four modalities of student learning that were described in a 1992 study by Fleming and Mills [20], i.e., visual, auditory, reading/writing, or kinesthetic. Each question is aimed at placing respondents in a “learning” situation. The respondents were permitted to choose two or more options if appropriate. The distribution of the VARK preferences was calculated according to the guidelines mentioned on the VARK website (https://vark-learn.com/). Accordingly, learning preferences were categorized as unimodal (V, A, R, or K), bimodal (VA, VR, AR, VK, AK, and RK), trimodal (VAR, ARK, VRK, and VAK), or quadmodal (VARK).
3. A yes/no question asking student satisfaction regarding current teaching modality in institute

All items were entered into Google Forms (Google LLC) and distributed online to students via WhatsApp, social media accounts of institutes, and email in three waves of invitation: wave 1 (1st May), wave 2 (1st June), and wave 3 (1st July 2021). Data collection was stopped on 20^th^ July due to time saturation.

### 2.3. Data Collection

Students at undergraduate medical and dental colleges, studying in private/public institutes of Pakistan, were invited to participate in the study through virtual snowballing technique. Educational outcome was calculated by GPA, accessed from students' academic record history by getting their student's allotted numbers. Students in Pakistan are awarded percentages instead of traditional GPA, so their percentages were converted into grades as follows: A (80-100%), B (70-79%), C (60-69%), and D (50-59%). Secondly, VARK questionnaire was asked. The student's educational outcome was also analyzed and correlated with the student's learning preferences. The sample was collected until time and data saturation was reached.

### 2.4. Data Analysis

The data was screened for inaccuracies. Forms with incomprehensible answers to open-ended questions (age, percentage obtained in last professional exam) were excluded. All responses on Google Forms were made mandatory to avoid missed data. Descriptive statistics were done on the overall sample [21]. All analyses were done using IBM SPSS statistical software, version 24 (IBM Corporation, New York, New York), and Microsoft Excel 2013 (Microsoft Corporation, Redmond, Washington). The Fisher test and chi-square test were used to compare the learning preferences between genders and public/private sector students and among preclinical/clinical years. Student characteristics were used as predictors for probability reporting of each learning style in comparison to unimodal style. A *p* value of less than 0.05 was considered significant.

## 3. Results

A total of 1473 out of 1651 students completed the questionnaire with 98.12% response rate with 42.9% male and 57% female respondents. The demographic data of respondents is represented in Table 1. In terms of academic rank, most of the students were of C grade (37.6%), followed by grade B (30%), A (27.5%), and lastly D (4.8%).

**Figure 1:**
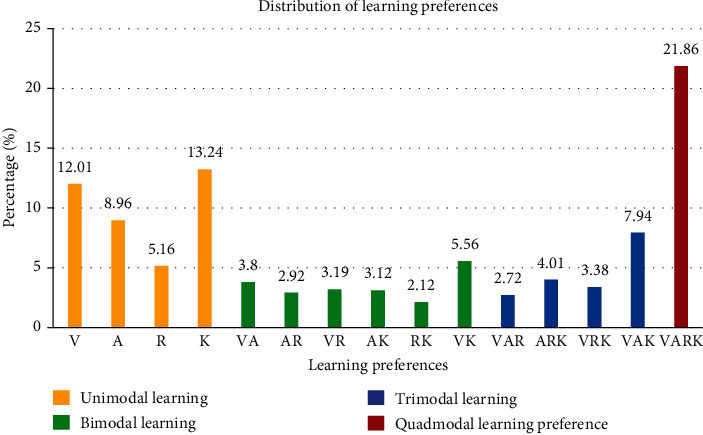
Overall distribution of learning preferences of respondents.

Of the study group, 39.37% (580) of the students preferred a unimodal learning style (either visual, auditory, reading/writing, or kinesthetic) and 60.62% (893) preferred multimodal style. Students had bimodal (20.71%), trimodal (18.05%), and quadmodal (21.86%) learning preferences, respectively. The most preferred unimodal learning style was kinesthetic (K), followed by visual (V), aural (A), and read/write (R). The most preferred bimodal style was VK (visual and kinesthetic), and the most preferred trimodal style was VAK (visual, aural, and kinesthetic) (Figure 1).

**Figure 2:**
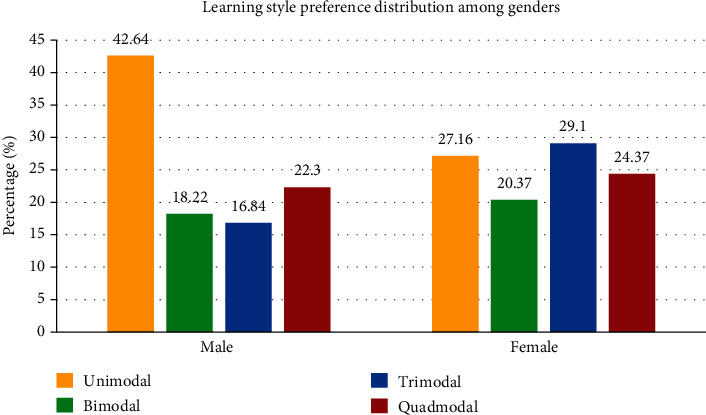
Learning style preference distribution among genders.

**Figure 3:**
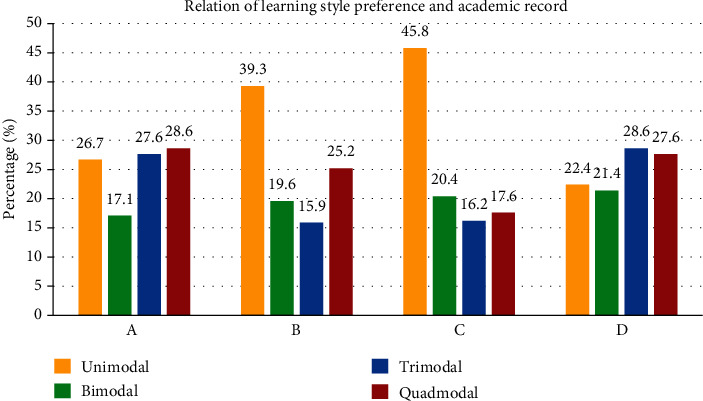
Learning style preferences of respondents according to their academic record.

Between genders, the multinomial regression revealed a statistically significant difference between male and female students. Male students had higher probability of choosing unimodal learning style (K) whereas female students had a higher probability of choosing trimodal (VAK) learning style (relative risk = 2.37) (Figure 2). When considering academic performance of students, C and D grade students are less likely to choose trimodal and quadmodal learning styles in comparison to A grade students (relative risk = 0.34) (Figure 3).

**Figure 4:**
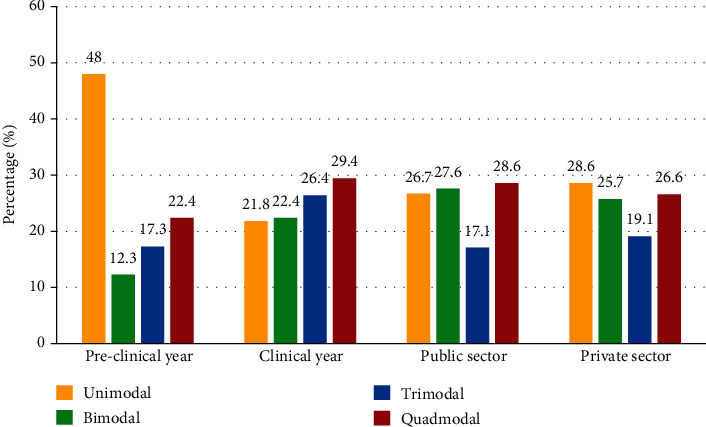
Learning style preference of students of preclinical/clinical years and of students from public/private sector institution.

Learning style preference of preclinical and clinical year students was also compared. Students of clinical years prefer kinesthetic (K) and visual (V) learning styles, whereas students of clinical year are more inclined towards audio (A) and visual (V) learning style. Students of preclinical year had lesser probability of choosing multimodal over unimodal when compared to students of clinical years (relative risk = 0.36) (Figure 4). There was no significant difference between choice of MBBS and BDS students. There was no significant difference between opinions of public sector and private sector students. Finally, when asked about the student satisfaction on current teaching methodology, an alarming 78% of respondents expressed dissatisfaction.

## 4. Discussion

Quite a number of studies have been done to investigate the learning style preferences of undergraduate medical and dental students, but all of those were conducted during face-to-face traditional teaching phase. Due to COVID-19, however, there has been a paradigm shift in the higher education of Pakistan forcing students and teachers to adapt online learning. To the best of our knowledge, this is the first study undertaken in the midst of technologically driven online education system.

Students were approached via social media websites, WhatsApp groups, and emails. Participation in the study is voluntary, and most of the participants were females. This is in contrast to several previous studies [22, 23]. This may be explained due to increased number of registered female students in medical and dental institutions in all provinces of Pakistan [24].

More than 60% students preferred multimodal learning style with no significant difference between bimodal, trimodal, and quadmodal preferences. Results of previous studies are in agreement with multimodal style being the dominant learning preference [3, 5, 22]. Adult learners prefer to acquire knowledge through different modalities. With the incorporation of online learning in higher education, the role of multimedia has increased substantially. Cognitive load theory supports the fact that information acquired through various sensory inputs helps transfer of information into long-term memory [25]. A vast number of students preferred unimodal style among which kinesthetic (K) was most common followed by visual (V). This result differs with previous studies. In Barbados, students preferred read/write (R) followed by kinesthetic (K) learning style [5]. In Saudi Arabia, dental students preferred aural (A) followed by kinesthetic (K) learning styles [22]. Similar result was seen in Iranian students [2]. A previous study done in Pakistan revealed that the preferred learning style of medical students was kinesthetic (K) followed by aural (A) [26]. Students might have begun to rely on web-based tools to enhance their learning. Studies reveal that 83.9% of medical students use YouTube as a learning tool [27–29]. Kinesthetic (K) has been a preferred style in several studies [30, 31] which demonstrates that students prefer active learning strategies. The shift in the preference of medical and dental students predominantly from aural (A) to visual (V) could be due to the increased use of online learning. However, further studies are required to postulate a direct impact of online learning on learning preference style of these students. The field of healthcare requires critical thinking and problem-solving techniques to treat patients effectively. Kinesthetic style inculcates active thinking in students [32]. Similar reason could account for the difference in learning preference style of preclinical and clinical year students. Students in the preclinical years are not dealing with patients; they acquire and retain information for the sake of passing their academic year. Thus, they are content with a single learning style in comparison to clinical year students.

When considering association of genders with learning style preference, there was a significant difference between the styles of both. Male students were more likely to choose unimodal style (K), whereas female students were more likely to choose multimodal learning style. This result negates few earlier studies where male students preferred multimodal style more than female students [5, 33]. Some studies revealed no significant difference in gender learning style preference [34], and a few studies had similar results to ours [22, 35]. Female students perhaps depend on a variety of different styles because they are notably more creative and are not satisfied with a single modality, due to which they switch to alternate styles of learning for a single topic.

Medical education teaching in Pakistan has been the same for the past 50 years. Only recently, due to COVID-19, educators were forced to include online teaching component. We believe our study provides an insight to the evolved learning preference styles of medical and dental students. It also highlights the influence of gender, academic year, and academic record on learning preference styles of students. Health professional educators can use this information and modify their teaching design to better accommodate today's student. Similarly, students should also be encouraged to learn from diverse teaching styles.

Limitations of the study include the lack of generalizability due to low sample size. Our study was time-restrained, and thus, we stopped data collection due to time saturation. Follow-up studies with a larger sample size are required to obtain generalizability. Similarly, follow-up qualitative studies are required to further explore the reasons behind this change and to explore any cause-effect relationship between online teaching learning and learning preference style.

## 5. Conclusion

The majority of medical and dental undergraduate students prefer multimodal learning styles. Gender, academic year, and academic record have a direct influence on the preference style. There was no significant difference between preference styles of medical and dental students. Nor was there a significant difference between students of public and private sector. A vast majority of students are dissatisfied with their current teaching modalities in institution. With this insight in the era of online learning, teachers must take active steps in incorporating multiple teaching modalities. Students with unimodal style should try other modalities as well to enhance their educational experience.

## Figures and Tables

**Table 1:** Demographic distribution of respondents.

Category	Frequency			
	MBBS	BDS	Male	Female
Total participants (*N* = 1473)	981 (66.6%)	492 (33.4%)	633 (42.9%)	840 (57%)
Preclinical year (*N* = 917)	626 (63.8%)	291 (59.1%)	431 (68.1%)	486 (57.8%)
Clinical year (*N* = 556)	355 (36.2%)	201 (40.8%)	202 (31.9%)	354 (42.1%)
Public institute (*N* = 637)	402 (41%)	235 (47.7%)	263 (41.5%)	374 (44.5%)
Private institute (*N* = 836)	579 (59%)	257 (52.2%)	370 (58.5%)	466 (55.5%)

## Data Availability

The raw data obtained from the respondents is included within the supplementary information file (available here). All the data generated and analyzed in this study are available from the corresponding author on reasonable request.

## Supplementary Materials

Supplementary Materials The supplementary file consists of a tabulation of participant responses on the questionnaire. It contains the demographics of all participants: their gender, age, field of study, year of study, and the type of degree program. It also includes the extracted information of each participant's regarding learning style preference where “1” means the chosen learning style and “0” indicates the nonchosen learning style of each participant. Finally, the file includes academic grade of each participant and satisfaction towards online learning (yes/no).
