# Supplementary material for: Identification of Preferred Learning Style of Medical and Dental Students Using VARK Questionnaire
Source: Biomed Res Int. 2021 Oct 18;2021:4355158. doi: 10.1155/2021/4355158 (PMC8545508; doi:10.1155/2021/4355158)
Supplement: Supplementary Materials — The supplementary file consists of a tabulation of participant responses on the questionnaire. It contains the demographics of all participants: their gender, age, field of study, year of study, and the type of degree program. It also includes the extracted information of each participant's regarding learning style preference where “1” means the chosen learning style and “0” indicates the nonchosen learning style of each participant. Finally, the file includes academic grade of each participant and satisfaction towards online learning (yes/no). [file 4355158.f1.pdf]

Repondant number	Gender	Age	Speciality	Degree program	Year of study
1	M	20	MBBS	Private	4
2	F	20	MBBS	Private	1
3	M	20	MBBS	Private	2
4	M	21	MBBS	Private	2
5	M	23	MBBS	public	3
6	M	19	MBBS	public	4
7	F	23	MBBS	public	1
8	M	20	MBBS	public	2
9	F	21	MBBS	private	1
10	F	19	MBBS	private	3
11	F	19	MBBS	private	1
12	M	18	MBBS	pubic	4
13	F	19	MBBS	private	4
14	F	20	MBBS	private	5
15	M	20	MBBS	private	5
16	F	21	MBBS	private	5
17	F	20	MBBS	public	5
18	M	23	MBBS	public	2
19	M	22	MBBS	public	1
20	M	22	MBBS	public	1
21	F	21	MBBS	Private	3
22	M	20	MBBS	Private	2
23	F	20	MBBS	Private	3
24	F	21	MBBS	Private	4
25	F	19	MBBS	public	4
26	M	19	MBBS	public	1
27	F	19	MBBS	public	2
28	F	20	MBBS	public	5
29	M	23	MBBS	private	5
30	F	23	MBBS	private	2
31	F	20	MBBS	private	2
32	M	20	MBBS	pubic	3
33	M	20	MBBS	private	1
34	M	21	MBBS	private	2
35	F	23	MBBS	private	1
36	M	19	MBBS	private	2
37	F	23	MBBS	public	4
38	F	20	MBBS	public	5
39	F	21	MBBS	public	1
40	M	19	MBBS	public	4
41	F	19	MBBS	Private	5
42	F	18	MBBS	Private	1
43	M	19	MBBS	Private	4
44	F	20	MBBS	Private	2
45	F	20	MBBS	public	5

46	M	21	MBBS	public	3
47	M	20	MBBS	public	1
48	M	23	MBBS	public	2
49	F	22	MBBS	private	2
50	M	22	MBBS	private	3
51	F	21	MBBS	private	4
52	F	20	MBBS	pubic	1
53	F	20	MBBS	private	2
54	M	21	MBBS	private	1
55	F	19	MBBS	private	3
56	F	19	MBBS	private	1
57	M	19	MBBS	public	4
58	F	20	MBBS	public	4
59	F	23	MBBS	public	5
60	M	23	MBBS	public	5
61	M	20	MBBS	Private	5
62	M	20	MBBS	Private	5
63	F	20	MBBS	Private	2
64	M	21	MBBS	Private	1
65	F	23	MBBS	public	1
66	F	19	MBBS	public	3
67	F	23	MBBS	public	2
68	M	20	MBBS	public	3
69	F	21	MBBS	private	4
70	F	19	MBBS	private	4
71	M	19	MBBS	private	1
72	F	18	MBBS	pubic	2
73	F	19	MBBS	private	5
74	M	20	MBBS	private	5
75	M	20	MBBS	private	2
76	M	21	MBBS	private	2
77	F	20	MBBS	public	3
78	M	23	MBBS	public	1
79	F	22	MBBS	public	2
80	F	22	MBBS	public	1
81	F	21	MBBS	Private	2
82	M	20	MBBS	Private	4
83	F	20	MBBS	Private	5
84	F	21	MBBS	Private	1
85	M	19	MBBS	public	4
86	F	19	MBBS	public	5
87	F	19	MBBS	public	1
88	M	20	MBBS	public	4
89	M	23	MBBS	private	2
90	M	23	MBBS	private	5
91	F	20	MBBS	private	3
92	M	20	MBBS	pubic	1

93	F	20	MBBS	private	2
94	F	21	MBBS	private	2
95	F	23	MBBS	private	3
96	M	19	MBBS	private	4
97	F	23	MBBS	public	1
98	F	20	MBBS	public	2
99	M	21	MBBS	public	1
100	F	19	MBBS	public	3
101	F	19	MBBS	Private	1
102	M	18	MBBS	Private	4
103	M	19	MBBS	Private	4
104	M	20	MBBS	Private	5
105	F	20	MBBS	public	5
106	M	21	MBBS	public	5
107	F	20	MBBS	public	5
108	F	23	MBBS	public	2
109	F	22	MBBS	private	1
110	M	22	MBBS	private	1
111	F	21	MBBS	private	3
112	F	20	MBBS	pubic	2
113	M	20	MBBS	private	3
114	F	21	MBBS	private	4
115	F	19	MBBS	private	4
116	M	19	MBBS	private	1
117	M	19	MBBS	public	2
118	M	20	MBBS	public	5
119	F	23	MBBS	public	5
120	M	23	MBBS	public	2
121	F	20	MBBS	Private	2
122	F	20	MBBS	Private	3
123	F	20	MBBS	Private	1
124	M	21	MBBS	Private	2
125	F	23	MBBS	public	1
126	F	19	MBBS	public	2
127	M	23	MBBS	public	4
128	F	20	MBBS	public	5
129	F	21	MBBS	private	1
130	M	19	MBBS	private	4
131	M	19	MBBS	private	5
132	M	18	MBBS	pubic	1
133	F	19	MBBS	private	4
134	M	20	MBBS	private	2
135	F	20	MBBS	private	5
136	F	21	MBBS	private	3
137	F	20	MBBS	public	1
138	M	23	MBBS	public	2
139	F	22	MBBS	public	2

140	F	22	MBBS	public	3
141	M	21	MBBS	Private	4
142	F	20	MBBS	Private	1
143	F	20	MBBS	Private	2
144	M	21	MBBS	Private	1
145	M	19	MBBS	public	3
146	M	19	MBBS	public	1
147	F	19	MBBS	public	4
148	M	20	MBBS	public	4
149	F	23	MBBS	private	5
150	F	23	MBBS	private	5
151	F	20	MBBS	private	5
152	M	20	MBBS	pubic	5
153	F	20	MBBS	private	2
154	F	21	MBBS	private	1
155	M	23	MBBS	private	1
156	F	19	MBBS	private	3
157	F	23	MBBS	public	2
158	M	20	MBBS	public	3
159	M	21	MBBS	public	4
160	M	19	MBBS	public	4
161	F	19	MBBS	Private	1
162	M	18	MBBS	Private	2
163	F	19	MBBS	Private	5
164	F	20	MBBS	Private	5
165	F	20	MBBS	public	2
166	M	21	MBBS	public	2
167	F	20	MBBS	public	3
168	F	23	MBBS	public	1
169	M	22	MBBS	private	2
170	F	22	MBBS	private	1
171	F	21	MBBS	private	2
172	M	20	MBBS	pubic	4
173	M	20	MBBS	private	5
174	M	21	MBBS	private	1
175	F	19	MBBS	private	4
176	M	19	MBBS	private	5
177	F	19	MBBS	public	1
178	F	20	MBBS	public	4
179	F	23	MBBS	public	2
180	M	23	MBBS	public	5
181	F	20	MBBS	Private	3
182	F	20	MBBS	Private	1
183	M	20	MBBS	Private	2
184	F	21	MBBS	Private	2
185	F	23	MBBS	public	3
186	M	19	MBBS	public	4

187	M	23	MBBS	public	1
188	M	20	MBBS	public	2
189	F	21	MBBS	private	1
190	M	19	MBBS	private	3
191	F	19	MBBS	private	1
192	F	18	MBBS	pubic	4
193	F	19	MBBS	private	4
194	M	20	MBBS	private	5
195	F	20	MBBS	private	5
196	F	21	MBBS	private	5
197	M	20	MBBS	public	5
198	F	23	MBBS	public	2
199	F	22	MBBS	public	1
200	M	22	MBBS	public	1
201	M	21	MBBS	Private	3
202	M	20	MBBS	Private	2
203	F	20	MBBS	Private	3
204	M	21	MBBS	Private	4
205	F	19	MBBS	public	4
206	F	19	MBBS	public	1
207	F	19	MBBS	public	2
208	M	20	MBBS	public	5
209	F	23	MBBS	private	5
210	F	23	MBBS	private	2
211	M	20	MBBS	private	2
212	F	20	MBBS	pubic	3
213	F	20	MBBS	private	1
214	M	21	MBBS	private	2
215	M	23	MBBS	private	1
216	M	19	MBBS	private	2
217	F	23	MBBS	public	4
218	M	20	MBBS	public	5
219	F	21	MBBS	public	1
220	F	19	MBBS	public	4
221	F	19	MBBS	Private	5
222	M	18	MBBS	Private	1
223	F	19	MBBS	Private	4
224	F	20	MBBS	Private	2
225	M	20	MBBS	public	5
226	F	21	MBBS	public	3
227	F	20	MBBS	public	1
228	M	23	MBBS	public	2
229	M	22	MBBS	private	2
230	M	22	MBBS	private	3
231	F	21	MBBS	private	4
232	M	20	MBBS	pubic	1
233	F	20	MBBS	private	2

234	F	21	MBBS	private	1
235	F	19	MBBS	private	3
236	M	19	MBBS	private	1
237	F	19	MBBS	public	4
238	F	20	MBBS	public	4
239	M	23	MBBS	public	5
240	F	23	MBBS	public	5
241	F	20	MBBS	Private	5
242	M	20	MBBS	Private	5
243	M	20	MBBS	Private	2
244	M	21	MBBS	Private	1
245	F	23	MBBS	public	1
246	M	19	MBBS	public	3
247	F	23	MBBS	public	2
248	F	20	MBBS	public	3
249	F	21	MBBS	private	4
250	M	19	MBBS	private	4
251	F	19	MBBS	private	1
252	F	18	MBBS	pubic	2
253	M	19	MBBS	private	5
254	F	20	MBBS	private	5
255	F	20	MBBS	private	2
256	M	21	MBBS	private	2
257	M	20	MBBS	public	3
258	M	23	MBBS	public	1
259	F	22	MBBS	public	2
260	M	22	MBBS	public	1
261	F	21	MBBS	Private	2
262	F	20	MBBS	Private	4
263	F	20	MBBS	Private	5
264	M	21	MBBS	Private	1
265	F	19	MBBS	public	4
266	F	19	MBBS	public	5
267	M	19	MBBS	public	1
268	F	20	MBBS	public	4
269	F	23	MBBS	private	2
270	M	23	MBBS	private	5
271	M	20	MBBS	private	3
272	M	20	MBBS	pubic	1
273	F	20	MBBS	private	2
274	M	21	MBBS	private	2
275	F	23	MBBS	private	3
276	F	19	MBBS	private	4
277	F	23	MBBS	public	1
278	M	20	MBBS	public	2
279	F	21	MBBS	public	1
280	F	19	MBBS	public	3

281	M	19	MBBS	Private	1
282	F	18	MBBS	Private	4
283	F	19	MBBS	Private	4
284	M	20	MBBS	Private	5
285	M	20	MBBS	public	5
286	M	21	MBBS	public	5
287	F	20	MBBS	public	5
288	M	23	MBBS	public	2
289	F	22	MBBS	private	1
290	F	22	MBBS	private	1
291	F	21	MBBS	private	3
292	M	20	MBBS	pubic	2
293	F	20	MBBS	private	3
294	F	21	MBBS	private	4
295	M	19	MBBS	private	4
296	F	19	MBBS	private	1
297	F	19	MBBS	public	2
298	M	20	MBBS	public	5
299	M	23	MBBS	public	5
300	M	23	MBBS	public	2
301	F	20	MBBS	Private	2
302	M	20	MBBS	Private	3
303	F	20	MBBS	Private	1
304	F	21	MBBS	Private	2
305	F	23	MBBS	public	1
306	M	19	MBBS	public	2
307	F	23	MBBS	public	4
308	F	20	MBBS	public	5
309	M	21	MBBS	private	1
310	F	19	MBBS	private	4
311	F	19	MBBS	private	5
312	M	18	MBBS	pubic	1
313	M	19	MBBS	private	4
314	M	20	MBBS	private	2
315	F	20	MBBS	private	5
316	M	21	MBBS	private	3
317	F	20	MBBS	public	1
318	F	23	MBBS	public	2
319	F	22	MBBS	public	2
320	M	22	MBBS	public	3
321	F	21	MBBS	Private	4
322	F	20	MBBS	Private	1
323	M	20	MBBS	Private	2
324	F	21	MBBS	Private	1
325	F	19	MBBS	public	3
326	M	19	MBBS	public	1
327	M	19	MBBS	public	4

328	M	20	MBBS	public	4
329	F	23	MBBS	private	5
330	M	23	MBBS	private	5
331	F	20	MBBS	private	5
332	F	20	MBBS	pubic	5
333	F	20	MBBS	private	2
334	M	21	MBBS	private	1
335	F	23	MBBS	private	1
336	F	19	MBBS	private	3
337	M	23	MBBS	public	2
338	F	20	MBBS	public	3
339	F	21	MBBS	public	4
340	M	19	MBBS	public	4
341	M	19	MBBS	Private	1
342	M	18	MBBS	Private	2
343	F	19	MBBS	Private	5
344	M	20	MBBS	Private	5
345	F	20	MBBS	public	2
346	F	21	MBBS	public	2
347	F	20	MBBS	public	3
348	M	23	MBBS	public	1
349	F	22	MBBS	private	2
350	F	22	MBBS	private	1
351	M	21	MBBS	private	2
352	F	20	MBBS	pubic	4
353	F	20	MBBS	private	5
354	M	21	MBBS	private	1
355	M	19	MBBS	private	4
356	M	19	MBBS	private	5
357	F	19	MBBS	public	1
358	M	20	MBBS	public	4
359	F	23	MBBS	public	2
360	F	23	MBBS	public	5
361	F	20	MBBS	Private	3
362	M	20	MBBS	Private	1
363	F	20	MBBS	Private	2
364	F	21	MBBS	Private	2
365	M	23	MBBS	public	3
366	F	19	MBBS	public	4
367	F	23	MBBS	public	1
368	M	20	MBBS	public	2
369	M	21	MBBS	private	1
370	M	19	MBBS	private	3
371	F	19	MBBS	private	1
372	M	18	MBBS	pubic	4
373	F	19	MBBS	private	4
374	F	20	MBBS	private	5

375	F	20	MBBS	private	5
376	M	21	MBBS	private	5
377	F	20	MBBS	public	5
378	F	23	MBBS	public	2
379	M	22	MBBS	public	1
380	F	22	MBBS	public	1
381	F	21	MBBS	private	3
382	M	20	MBBS	private	2
383	M	20	MBBS	private	3
384	M	21	MBBS	pubic	4
385	F	19	MBBS	private	4
386	M	19	MBBS	private	1
387	F	19	MBBS	private	2
388	F	20	MBBS	private	5
389	F	23	MBBS	public	5
390	M	23	MBBS	public	2
391	F	20	MBBS	public	2
392	F	20	MBBS	public	3
393	M	20	MBBS	Private	1
394	F	21	MBBS	Private	2
395	F	23	MBBS	Private	1
396	M	19	MBBS	Private	2
397	M	23	MBBS	public	4
398	M	20	MBBS	public	5
399	F	21	MBBS	public	1
400	M	19	MBBS	public	4
401	F	19	MBBS	private	5
402	F	18	MBBS	private	1
403	F	19	MBBS	private	4
404	M	20	MBBS	pubic	2
405	F	20	MBBS	private	5
406	F	21	MBBS	private	3
407	M	20	MBBS	private	1
408	F	23	MBBS	private	2
409	F	22	MBBS	public	2
410	M	22	MBBS	public	3
411	M	21	MBBS	public	4
412	M	20	MBBS	public	1
413	F	20	MBBS	Private	2
414	M	21	MBBS	Private	1
415	F	19	MBBS	Private	3
416	F	19	MBBS	Private	1
417	F	19	MBBS	public	4
418	M	20	MBBS	public	4
419	F	23	MBBS	public	5
420	F	23	MBBS	public	5
421	M	20	MBBS	private	5

422	F	20	MBBS	private	5
423	F	20	MBBS	private	2
424	M	21	MBBS	pubic	1
425	M	23	MBBS	private	1
426	M	19	MBBS	private	3
427	F	23	MBBS	private	2
428	M	20	MBBS	private	3
429	F	21	MBBS	public	4
430	F	19	MBBS	public	4
431	F	19	MBBS	public	1
432	M	18	MBBS	public	2
433	F	19	MBBS	Private	5
434	F	20	MBBS	Private	5
435	M	20	MBBS	Private	2
436	F	21	MBBS	Private	2
437	F	20	MBBS	public	3
438	M	23	MBBS	public	1
439	M	22	MBBS	public	2
440	M	22	MBBS	public	1
441	F	21	MBBS	private	2
442	M	20	MBBS	private	4
443	F	20	MBBS	private	5
444	F	21	MBBS	pubic	1
445	F	19	MBBS	private	4
446	M	19	MBBS	private	5
447	F	19	MBBS	private	1
448	F	20	MBBS	private	4
449	M	23	MBBS	public	2
450	F	23	MBBS	public	5
451	F	20	MBBS	public	3
452	M	20	MBBS	public	1
453	M	20	MBBS	Private	2
454	M	21	MBBS	Private	2
455	F	23	MBBS	Private	3
456	M	19	MBBS	Private	4
457	F	23	MBBS	public	1
458	F	20	MBBS	public	2
459	F	21	MBBS	public	1
460	M	19	MBBS	public	3
461	F	19	MBBS	private	1
462	F	18	MBBS	private	4
463	M	19	MBBS	private	4
464	F	20	MBBS	pubic	5
465	F	20	MBBS	private	5
466	M	21	MBBS	private	5
467	M	20	MBBS	private	5
468	M	23	MBBS	private	2

469	F	22	MBBS	public	1
470	M	22	MBBS	public	1
471	F	21	MBBS	public	3
472	F	20	MBBS	public	2
473	F	20	MBBS	Private	3
474	M	21	MBBS	Private	4
475	F	19	MBBS	Private	4
476	F	19	MBBS	Private	1
477	M	19	MBBS	public	2
478	F	20	MBBS	public	5
479	F	23	MBBS	public	5
480	M	23	MBBS	public	2
481	M	20	MBBS	private	2
482	M	20	MBBS	private	3
483	F	20	MBBS	private	1
484	M	21	MBBS	pubic	2
485	F	23	MBBS	private	1
486	F	19	MBBS	private	2
487	F	23	MBBS	private	4
488	M	20	MBBS	private	5
489	F	21	MBBS	public	1
490	F	19	MBBS	public	4
491	M	19	MBBS	public	5
492	F	18	MBBS	public	1
493	F	19	MBBS	Private	4
494	M	20	MBBS	Private	2
495	M	20	MBBS	Private	5
496	M	21	MBBS	Private	3
497	F	20	MBBS	public	1
498	M	23	MBBS	public	2
499	F	22	MBBS	public	2
500	F	22	MBBS	public	3
501	F	21	MBBS	private	4
502	M	20	MBBS	private	1
503	F	20	MBBS	private	2
504	F	21	MBBS	pubic	1
505	M	19	MBBS	private	3
506	F	19	MBBS	private	1
507	F	19	MBBS	private	4
508	M	20	MBBS	private	4
509	M	23	MBBS	public	5
510	M	23	MBBS	public	5
511	F	20	MBBS	public	5
512	M	20	MBBS	public	5
513	F	20	MBBS	Private	2
514	F	21	MBBS	Private	1
515	F	23	MBBS	Private	1

516	M	19	MBBS	Private	3
517	F	23	MBBS	public	2
518	F	20	MBBS	public	3
519	M	21	MBBS	public	4
520	F	19	MBBS	public	4
521	F	19	MBBS	private	1
522	M	18	MBBS	private	2
523	M	19	MBBS	private	5
524	M	20	MBBS	pubic	5
525	F	20	MBBS	private	2
526	M	21	MBBS	private	2
527	F	20	MBBS	private	3
528	F	23	MBBS	private	1
529	F	22	MBBS	public	2
530	M	22	MBBS	public	1
531	F	21	MBBS	public	2
532	F	20	MBBS	public	4
533	M	20	MBBS	Private	5
534	F	21	MBBS	Private	1
535	F	19	MBBS	Private	4
536	M	19	MBBS	Private	5
537	M	19	MBBS	public	1
538	M	20	MBBS	public	4
539	F	23	MBBS	public	2
540	M	23	MBBS	public	5
541	F	20	MBBS	private	3
542	F	20	MBBS	private	1
543	F	20	MBBS	private	2
544	M	21	MBBS	pubic	2
545	F	23	MBBS	private	3
546	F	19	MBBS	private	4
547	M	23	MBBS	private	1
548	F	20	MBBS	private	2
549	F	21	MBBS	public	1
550	M	19	MBBS	public	3
551	M	19	MBBS	public	1
552	M	18	MBBS	public	4
553	F	19	MBBS	Private	4
554	M	20	MBBS	Private	5
555	F	20	MBBS	Private	5
556	F	21	MBBS	Private	5
557	F	20	MBBS	public	5
558	M	23	MBBS	public	2
559	F	22	MBBS	public	1
560	F	22	MBBS	public	1
561	M	21	MBBS	private	3
562	F	20	MBBS	private	2

563	F	20	MBBS	private	3
564	M	21	MBBS	pubic	4
565	M	19	MBBS	private	4
566	M	19	MBBS	private	1
567	F	19	MBBS	private	2
568	M	20	MBBS	private	5
569	F	23	MBBS	public	5
570	F	23	MBBS	public	2
571	F	20	MBBS	public	2
572	M	20	MBBS	public	3
573	F	20	MBBS	Private	1
574	F	21	MBBS	Private	2
575	M	23	MBBS	Private	1
576	F	19	MBBS	Private	2
577	F	23	MBBS	public	4
578	M	20	MBBS	public	5
579	M	21	MBBS	public	1
580	M	19	MBBS	public	4
581	F	19	MBBS	private	5
582	M	18	MBBS	private	1
583	F	19	MBBS	private	4
584	F	20	MBBS	pubic	2
585	F	20	MBBS	private	5
586	M	21	MBBS	private	3
587	F	20	MBBS	private	1
588	F	23	MBBS	private	2
589	M	22	MBBS	public	2
590	F	22	MBBS	public	3
591	F	21	MBBS	public	4
592	M	20	MBBS	public	1
593	M	20	MBBS	Private	2
594	M	21	MBBS	Private	1
595	F	19	MBBS	Private	3
596	M	19	MBBS	Private	1
597	F	19	MBBS	public	4
598	F	20	MBBS	public	4
599	F	23	MBBS	public	5
600	M	23	MBBS	public	5
601	F	20	MBBS	private	5
602	F	20	MBBS	private	5
603	M	20	MBBS	private	2
604	F	21	MBBS	pubic	1
605	F	23	MBBS	private	1
606	M	19	MBBS	private	3
607	M	23	MBBS	private	2
608	M	20	MBBS	private	3
609	F	21	MBBS	public	4

610	M	19	MBBS	public	4
611	F	19	MBBS	public	1
612	F	18	MBBS	public	2
613	F	19	MBBS	Private	5
614	M	20	MBBS	Private	5
615	F	20	MBBS	Private	2
616	F	21	MBBS	Private	2
617	M	20	MBBS	public	3
618	F	23	MBBS	public	1
619	F	22	MBBS	public	2
620	M	22	MBBS	public	1
621	M	21	MBBS	private	2
622	M	20	MBBS	private	4
623	F	20	MBBS	private	5
624	M	21	MBBS	pubic	1
625	F	19	MBBS	private	4
626	F	19	MBBS	private	5
627	F	19	MBBS	private	1
628	M	20	MBBS	private	4
629	F	23	MBBS	public	2
630	F	23	MBBS	public	5
631	M	20	MBBS	public	3
632	F	20	MBBS	public	1
633	F	20	MBBS	Private	2
634	M	21	MBBS	Private	2
635	M	23	MBBS	Private	3
636	M	19	MBBS	Private	4
637	F	23	MBBS	public	1
638	M	20	MBBS	public	2
639	F	21	MBBS	public	1
640	F	19	MBBS	public	3
641	F	19	MBBS	private	1
642	M	18	MBBS	private	4
643	F	19	MBBS	private	4
644	F	20	MBBS	pubic	5
645	M	20	MBBS	private	5
646	F	21	MBBS	private	5
647	F	20	MBBS	private	5
648	M	23	MBBS	private	2
649	M	22	MBBS	public	1
650	M	22	MBBS	public	1
651	F	21	MBBS	public	3
652	M	20	MBBS	public	2
653	F	20	MBBS	private	3
654	F	21	MBBS	private	4
655	F	19	MBBS	private	4
656	M	19	MBBS	pubic	1

657	F	19	MBBS	private	2
658	F	20	MBBS	private	5
659	M	23	MBBS	private	5
660	F	23	MBBS	private	2
661	F	20	MBBS	public	2
662	M	20	MBBS	public	3
663	M	20	MBBS	public	1
664	M	21	MBBS	public	2
665	F	23	MBBS	Private	1
666	M	19	MBBS	Private	2
667	F	23	MBBS	Private	4
668	F	20	MBBS	Private	5
669	F	21	MBBS	public	1
670	M	19	MBBS	public	4
671	F	19	MBBS	public	5
672	F	18	MBBS	public	1
673	M	19	MBBS	private	4
674	F	20	MBBS	private	2
675	F	20	MBBS	private	5
676	M	21	MBBS	pubic	3
677	M	20	MBBS	private	1
678	M	23	MBBS	private	2
679	F	22	MBBS	private	2
680	M	22	MBBS	private	3
681	F	21	MBBS	public	4
682	F	20	MBBS	public	1
683	F	20	MBBS	public	2
684	M	21	MBBS	public	1
685	F	19	MBBS	Private	3
686	F	19	MBBS	Private	1
687	M	19	MBBS	Private	4
688	F	20	MBBS	Private	4
689	F	23	MBBS	public	5
690	M	23	MBBS	public	5
691	M	23	MBBS	public	5
692	M	20	MBBS	public	5
693	F	20	MBBS	private	2
694	M	20	MBBS	private	1
695	F	21	MBBS	private	1
696	F	23	MBBS	pubic	3
697	F	19	MBBS	private	2
698	M	23	MBBS	private	3
699	F	20	MBBS	private	4
700	F	21	MBBS	private	4
701	M	19	MBBS	public	1
702	F	19	MBBS	public	2
703	F	18	MBBS	public	5

704	M	19	MBBS	public	5
705	M	20	MBBS	Private	2
706	M	20	MBBS	Private	2
707	F	21	MBBS	Private	3
708	M	20	MBBS	Private	1
709	F	23	MBBS	public	2
710	F	22	MBBS	public	1
711	F	22	MBBS	public	2
712	M	21	MBBS	public	4
713	F	20	MBBS	private	5
714	F	20	MBBS	private	1
715	M	21	MBBS	private	4
716	F	19	MBBS	pubic	5
717	F	19	MBBS	private	1
718	M	19	MBBS	private	4
719	M	20	MBBS	private	2
720	M	23	MBBS	private	5
721	F	23	MBBS	public	3
722	M	20	MBBS	public	1
723	F	20	MBBS	public	2
724	F	20	MBBS	public	2
725	F	21	MBBS	Private	3
726	M	23	MBBS	Private	4
727	F	19	MBBS	Private	1
728	F	23	MBBS	Private	2
729	M	20	MBBS	public	1
730	F	21	MBBS	public	3
731	F	19	MBBS	public	1
732	M	19	MBBS	public	4
733	M	18	MBBS	private	4
734	M	19	MBBS	private	5
735	F	20	MBBS	private	5
736	M	20	MBBS	pubic	5
737	F	21	MBBS	private	5
738	F	20	MBBS	private	2
739	F	23	MBBS	private	1
740	M	22	MBBS	private	1
741	F	22	MBBS	public	3
742	F	21	MBBS	public	2
743	M	20	MBBS	public	3
744	F	20	MBBS	public	4
745	F	21	MBBS	Private	4
746	M	19	MBBS	Private	1
747	M	19	MBBS	Private	2
748	M	19	MBBS	Private	5
749	F	20	MBBS	public	5
750	M	23	MBBS	public	2

751	F	23	MBBS	public	2
752	F	20	MBBS	public	3
753	F	20	MBBS	private	1
754	M	20	MBBS	private	2
755	F	21	MBBS	private	1
756	F	23	MBBS	pubic	2
757	M	19	MBBS	private	4
758	F	23	MBBS	private	5
759	F	20	MBBS	private	1
760	M	21	MBBS	private	4
761	M	19	MBBS	public	5
762	M	19	MBBS	public	1
763	F	18	MBBS	public	4
764	M	19	MBBS	public	2
765	F	20	MBBS	Private	5
766	F	20	MBBS	Private	3
767	F	21	MBBS	Private	1
768	M	20	MBBS	Private	2
769	F	23	MBBS	public	2
770	F	22	MBBS	public	3
771	M	22	MBBS	public	4
772	F	21	MBBS	public	1
773	F	20	MBBS	private	2
774	M	20	MBBS	private	1
775	M	21	MBBS	private	3
776	M	19	MBBS	pubic	1
777	F	19	MBBS	private	4
778	M	19	MBBS	private	4
779	F	20	MBBS	private	5
780	F	23	MBBS	private	5
781	F	23	MBBS	public	5
782	M	20	MBBS	public	5
783	F	20	MBBS	public	2
784	F	20	MBBS	public	1
785	M	21	MBBS	Private	1
786	F	23	MBBS	Private	3
787	F	19	MBBS	Private	2
788	M	23	MBBS	Private	3
789	M	20	MBBS	public	4
790	M	21	MBBS	public	4
791	F	19	MBBS	public	1
792	M	19	MBBS	public	2
793	F	18	MBBS	private	5
794	F	19	MBBS	private	5
795	F	20	MBBS	private	2
796	M	20	MBBS	pubic	2
797	F	21	MBBS	private	3

798	F	20	MBBS	private	1
799	M	23	MBBS	private	2
800	F	22	MBBS	private	1
801	F	22	MBBS	public	2
802	M	21	MBBS	public	4
803	M	20	MBBS	public	5
804	M	20	MBBS	public	1
805	F	21	MBBS	Private	4
806	M	19	MBBS	Private	5
807	F	19	MBBS	Private	1
808	F	19	MBBS	Private	4
809	F	20	MBBS	public	2
810	M	23	MBBS	public	5
811	F	23	MBBS	public	3
812	F	20	MBBS	public	1
813	M	20	MBBS	private	2
814	F	20	MBBS	private	2
815	F	21	MBBS	private	3
816	M	23	MBBS	pubic	4
817	M	19	MBBS	private	1
818	M	23	MBBS	private	2
819	F	20	MBBS	private	1
820	M	21	MBBS	private	3
821	F	19	MBBS	public	1
822	F	19	MBBS	public	4
823	F	18	MBBS	public	4
824	M	19	MBBS	public	5
825	F	20	MBBS	Private	5
826	F	20	MBBS	Private	5
827	M	21	MBBS	Private	5
828	F	20	MBBS	Private	2
829	F	23	MBBS	public	1
830	M	22	MBBS	public	1
831	M	22	MBBS	public	3
832	M	21	MBBS	public	2
833	F	20	MBBS	private	3
834	M	20	MBBS	private	4
835	F	21	MBBS	private	4
836	F	19	MBBS	pubic	1
837	F	19	MBBS	private	2
838	M	19	MBBS	private	5
839	F	20	MBBS	private	5
840	F	23	MBBS	private	2
841	M	23	MBBS	public	2
842	F	20	MBBS	public	3
843	F	20	MBBS	public	1
844	M	20	MBBS	public	2

845	M	21	MBBS	Private	1
846	M	23	MBBS	Private	2
847	F	19	MBBS	Private	4
848	M	23	MBBS	Private	5
849	F	20	MBBS	public	1
850	F	21	MBBS	public	4
851	F	19	MBBS	public	5
852	M	19	MBBS	public	1
853	F	18	MBBS	private	4
854	F	19	MBBS	private	2
855	M	20	MBBS	private	5
856	F	20	MBBS	pubic	3
857	F	21	MBBS	private	1
858	M	20	MBBS	private	2
859	M	23	MBBS	private	2
860	M	22	MBBS	private	3
861	F	22	MBBS	public	4
862	M	21	MBBS	public	1
863	F	20	MBBS	public	2
864	F	20	MBBS	public	1
865	F	21	MBBS	Private	3
866	M	19	MBBS	Private	1
867	F	19	MBBS	Private	4
868	F	19	MBBS	Private	4
869	M	20	MBBS	public	5
870	F	23	MBBS	public	5
871	F	23	MBBS	public	5
872	M	20	MBBS	public	5
873	M	20	MBBS	private	2
874	M	20	MBBS	private	1
875	F	21	MBBS	private	1
876	M	23	MBBS	pubic	3
877	F	19	MBBS	private	2
878	F	23	MBBS	private	3
879	F	20	MBBS	private	4
880	M	21	MBBS	private	4
881	F	19	MBBS	public	1
882	F	19	MBBS	public	2
883	M	18	MBBS	public	5
884	F	19	MBBS	public	5
885	F	20	MBBS	Private	2
886	M	20	MBBS	Private	2
887	M	21	MBBS	Private	3
888	M	20	MBBS	Private	1
889	F	23	MBBS	public	2
890	M	22	MBBS	public	1
891	F	22	MBBS	public	2

892	F	21	MBBS	public	4
893	F	20	MBBS	private	5
894	M	20	MBBS	private	1
895	F	21	MBBS	private	4
896	F	19	MBBS	pubic	5
897	M	19	MBBS	private	1
898	F	19	MBBS	private	4
899	F	20	MBBS	private	2
900	M	23	MBBS	private	5
901	M	23	MBBS	public	3
902	M	20	MBBS	public	1
903	F	20	MBBS	public	2
904	M	20	MBBS	public	2
905	F	21	MBBS	Private	3
906	F	23	MBBS	Private	4
907	F	19	MBBS	Private	1
908	M	23	MBBS	Private	2
909	F	20	MBBS	public	1
910	F	21	MBBS	public	3
911	M	19	MBBS	public	1
912	F	19	MBBS	public	4
913	F	18	MBBS	private	4
914	M	19	MBBS	private	5
915	M	20	MBBS	private	5
916	M	20	MBBS	pubic	5
917	F	21	MBBS	private	5
918	M	20	MBBS	private	2
919	F	23	MBBS	private	1
920	F	22	MBBS	private	1
921	F	22	MBBS	public	3
922	M	21	MBBS	public	2
923	F	20	MBBS	public	3
924	F	20	MBBS	public	4
925	M	21	MBBS	private	4
926	F	19	MBBS	private	1
927	F	19	MBBS	private	2
928	M	19	MBBS	pubic	5
929	M	20	MBBS	private	5
930	M	23	MBBS	private	2
931	F	23	MBBS	private	2
932	M	20	MBBS	private	3
933	F	20	MBBS	public	1
934	F	20	MBBS	public	2
935	F	21	MBBS	public	1
936	M	23	MBBS	public	2
937	F	19	MBBS	Private	4
938	F	23	MBBS	Private	5

939	M	20	MBBS	Private	1
940	F	21	MBBS	Private	4
941	F	19	MBBS	public	5
942	M	19	MBBS	public	1
943	M	18	MBBS	public	4
944	M	19	MBBS	public	2
945	F	20	MBBS	private	5
946	M	20	MBBS	private	3
947	F	21	MBBS	private	1
948	F	20	MBBS	pubic	2
949	F	23	MBBS	private	2
950	M	22	MBBS	private	3
951	F	22	MBBS	private	4
952	F	21	MBBS	private	1
953	M	20	MBBS	public	2
954	F	20	MBBS	public	1
955	F	21	MBBS	public	3
956	M	19	MBBS	public	1
957	M	19	MBBS	Private	4
958	M	19	MBBS	Private	4
959	F	20	MBBS	Private	5
960	M	23	MBBS	Private	5
961	F	23	MBBS	public	5
962	F	20	MBBS	public	5
963	F	20	MBBS	public	2
964	M	20	MBBS	public	1
965	F	21	MBBS	private	1
966	F	23	MBBS	private	3
967	M	19	MBBS	private	2
968	F	23	MBBS	pubic	3
969	F	20	MBBS	private	4
970	M	21	MBBS	private	4
971	M	19	MBBS	private	1
972	M	19	MBBS	private	2
973	F	18	MBBS	public	5
974	M	19	MBBS	public	5
975	F	20	MBBS	public	2
976	F	20	MBBS	public	2
977	F	21	MBBS	Private	3
978	M	20	MBBS	Private	1
979	F	23	MBBS	Private	2
980	F	22	MBBS	Private	1
981	M	22	MBBS	public	2
982	F	21	BDS	public	4
983	F	20	BDS	public	4
984	M	20	BDS	public	1
985	M	21	BDS	private	4

986	M	19	BDS	private	2
987	F	19	BDS	private	1
988	M	19	BDS	pubic	4
989	F	20	BDS	private	2
990	F	23	BDS	private	2
991	F	23	BDS	private	3
992	M	20	BDS	private	4
993	F	20	BDS	public	4
994	F	20	BDS	public	1
995	M	21	BDS	public	4
996	F	23	BDS	public	2
997	F	19	BDS	Private	1
998	M	23	BDS	Private	4
999	M	20	BDS	Private	2
1000	M	21	BDS	Private	2
1001	F	19	BDS	public	3
1002	M	19	BDS	public	4
1003	F	18	BDS	public	4
1004	F	19	BDS	public	1
1005	F	20	BDS	private	4
1006	M	20	BDS	private	2
1007	F	21	BDS	private	1
1008	F	20	BDS	pubic	4
1009	M	23	BDS	private	2
1010	F	22	BDS	private	2
1011	F	22	BDS	private	3
1012	M	21	BDS	private	4
1013	M	20	BDS	public	2
1014	M	20	BDS	public	1
1015	F	21	BDS	public	4
1016	M	19	BDS	public	2
1017	F	19	BDS	Private	1
1018	F	19	BDS	Private	4
1019	F	20	BDS	Private	2
1020	M	23	BDS	Private	2
1021	F	23	BDS	public	3
1022	F	20	BDS	public	4
1023	M	20	BDS	public	1
1024	F	20	BDS	public	4
1025	F	21	BDS	private	2
1026	M	23	BDS	private	1
1027	M	19	BDS	private	4
1028	M	23	BDS	pubic	2
1029	F	20	BDS	private	2
1030	M	21	BDS	private	3
1031	F	19	BDS	private	4
1032	F	19	BDS	private	4

1033	F	18	BDS	public	1
1034	M	19	BDS	public	4
1035	F	20	BDS	public	2
1036	F	20	BDS	public	1
1037	M	21	BDS	Private	4
1038	F	20	BDS	Private	2
1039	F	23	BDS	Private	2
1040	M	22	BDS	Private	3
1041	M	22	BDS	public	4
1042	M	21	BDS	public	4
1043	F	20	BDS	public	1
1044	M	20	BDS	public	4
1045	F	21	BDS	private	2
1046	F	19	BDS	private	1
1047	F	19	BDS	private	4
1048	M	19	BDS	pubic	2
1049	F	20	BDS	private	2
1050	F	23	BDS	private	3
1051	M	23	BDS	private	4
1052	F	20	BDS	private	2
1053	F	20	BDS	public	1
1054	M	20	BDS	public	4
1055	M	21	BDS	public	2
1056	M	23	BDS	public	1
1057	F	19	BDS	Private	4
1058	M	23	BDS	Private	2
1059	F	20	BDS	Private	2
1060	F	21	BDS	Private	3
1061	F	19	BDS	public	4
1062	M	19	BDS	public	1
1063	F	18	BDS	public	4
1064	F	19	BDS	public	2
1065	M	20	BDS	private	1
1066	F	20	BDS	private	4
1067	F	21	BDS	private	2
1068	M	20	BDS	pubic	2
1069	M	23	BDS	private	3
1070	M	22	BDS	private	4
1071	F	22	BDS	private	4
1072	M	21	BDS	private	1
1073	F	20	BDS	public	4
1074	F	20	BDS	public	2
1075	F	21	BDS	public	1
1076	M	19	BDS	public	4
1077	F	19	BDS	Private	2
1078	F	19	BDS	Private	2
1079		20	BDS	Private	3

1080	F	23	BDS	Private	4
1081	M	23	BDS	public	4
1082	F	20	BDS	public	1
1083	F	20	BDS	public	4
1084	M	20	BDS	public	2
1085	F	21	BDS	private	1
1086	F	23	BDS	private	4
1087	M	19	BDS	private	2
1088	M	23	BDS	pubic	2
1089	M	20	BDS	private	3
1090	F	21	BDS	private	4
1091	M	19	BDS	private	2
1092	F	19	BDS	private	1
1093	F	18	BDS	public	4
1094	F	19	BDS	public	2
1095	M	20	BDS	public	1
1096	F	20	BDS	public	4
1097	F	21	BDS	Private	2
1098	M	20	BDS	Private	2
1099	F	23	BDS	Private	3
1100	F	22	BDS	Private	4
1101	M	22	BDS	public	1
1102	M	21	BDS	public	4
1103	M	20	BDS	public	2
1104	F	20	BDS	public	1
1105	M	21	BDS	private	4
1106	F	19	BDS	private	2
1107	F	19	BDS	private	2
1108	F	19	BDS	pubic	3
1109	M	20	BDS	private	4
1110	F	23	BDS	private	4
1111	F	23	BDS	private	1
1112	M	20	BDS	private	4
1113	F	20	BDS	public	2
1114	F	20	BDS	public	1
1115	M	21	BDS	public	4
1116	M	23	BDS	public	2
1117	M	19	BDS	Private	2
1118	F	23	BDS	Private	3
1119	M	20	BDS	Private	4
1120	F	21	BDS	Private	4
1121	F	19	BDS	public	1
1122	F	19	BDS	public	4
1123	M	18	BDS	public	2
1124	F	19	BDS	public	1
1125	F	20	BDS	private	4
1126	M	20	BDS	private	2

1127	F	21	BDS	private	2
1128	F	20	BDS	pubic	3
1129	M	23	BDS	private	4
1130	M	22	BDS	private	2
1131	M	22	BDS	private	1
1132	F	21	BDS	private	4
1133	M	20	BDS	public	2
1134	F	20	BDS	public	1
1135	F	21	BDS	public	4
1136	F	19	BDS	public	2
1137	M	19	BDS	Private	2
1138	F	19	BDS	Private	3
1139	F	20	BDS	Private	4
1140	M	23	BDS	Private	1
1141	F	23	BDS	public	4
1142	F	20	BDS	public	2
1143	M	20	BDS	public	1
1144	M	20	BDS	public	4
1145	M	21	BDS	private	2
1146	F	23	BDS	private	2
1147	M	19	BDS	private	3
1148	F	23	BDS	pubic	4
1149	F	20	BDS	private	4
1150	F	21	BDS	private	1
1151	M	19	BDS	private	4
1152	F	19	BDS	private	2
1153	F	18	BDS	public	1
1154	M	19	BDS	public	4
1155	F	20	BDS	public	2
1156	F	20	BDS	public	2
1157	M	21	BDS	Private	3
1158	M	20	BDS	Private	4
1159	M	23	BDS	Private	4
1160	F	22	BDS	Private	1
1161	M	22	BDS	public	4
1162	F	21	BDS	public	2
1163	F	20	BDS	public	1
1164	F	20	BDS	public	4
1165	M	21	BDS	private	2
1166	F	19	BDS	private	2
1167	F	19	BDS	private	3
1168	M	19	BDS	pubic	4
1169	F	20	BDS	private	2
1170	F	23	BDS	private	1
1171	M	23	BDS	private	4
1172	M	20	BDS	private	2
1173	M	20	BDS	public	1

1174	F	20	BDS	public	4
1175	M	21	BDS	public	2
1176	F	23	BDS	public	2
1177	F	19	BDS	Private	3
1178	F	23	BDS	Private	4
1179	M	20	BDS	Private	1
1180	F	21	BDS	Private	4
1181	F	19	BDS	public	2
1182	M	19	BDS	public	1
1183	F	18	BDS	public	4
1184	F	19	BDS	public	2
1185	M	20	BDS	private	2
1186	M	20	BDS	private	3
1187	M	21	BDS	private	4
1188	F	20	BDS	pubic	4
1189	M	23	BDS	private	1
1190	F	22	BDS	private	4
1191	F	22	BDS	private	2
1192	F	21	BDS	private	1
1193	M	20	BDS	public	4
1194	F	20	BDS	public	2
1195	F	21	BDS	public	2
1196		19	BDS	public	3
1197	F	19	BDS	private	4
1198	M	19	BDS	private	4
1199	F	20	BDS	private	1
1200	F	23	BDS	pubic	4
1201	M	23	BDS	private	2
1202	F	20	BDS	private	1
1203	F	20	BDS	private	4
1204	M	20	BDS	private	2
1205	M	21	BDS	public	2
1206	M	23	BDS	public	3
1207	F	19	BDS	public	4
1208	M	23	BDS	public	2
1209	F	20	BDS	Private	1
1210	F	21	BDS	Private	4
1211	F	19	BDS	Private	2
1212	M	19	BDS	Private	1
1213	F	18	BDS	public	4
1214	F	19	BDS	public	2
1215	M	20	BDS	public	2
1216	F	20	BDS	public	3
1217	F	21	BDS	private	4
1218	M	20	BDS	private	1
1219	M	23	BDS	private	4
1220	M	22	BDS	pubic	2

1221	F	22	BDS	private	1
1222	M	21	BDS	private	4
1223	F	20	BDS	private	2
1224	F	20	BDS	private	2
1225	F	21	BDS	public	3
1226	M	19	BDS	public	4
1227	F	19	BDS	public	4
1228	F	19	BDS	public	1
1229	M	20	BDS	Private	4
1230	F	23	BDS	Private	2
1231	F	23	BDS	Private	1
1232	M	20	BDS	Private	4
1233	M	20	BDS	public	2
1234	M	20	BDS	public	2
1235	F	21	BDS	public	3
1236	M	23	BDS	public	4
1237	F	19	BDS	private	4
1238	F	23	BDS	private	1
1239	F	20	BDS	private	4
1240	M	21	BDS	pubic	2
1241	F	19	BDS	private	1
1242	F	19	BDS	private	4
1243	M	18	BDS	private	2
1244	F	19	BDS	private	2
1245	F	20	BDS	public	3
1246	M	20	BDS	public	4
1247	M	21	BDS	public	2
1248	M	20	BDS	public	1
1249	F	23	BDS	Private	4
1250	M	22	BDS	Private	2
1251	F	22	BDS	Private	1
1252	F	21	BDS	Private	4
1253	F	20	BDS	public	2
1254	M	20	BDS	public	2
1255	F	21	BDS	public	3
1256	F	19	BDS	public	4
1257	M	19	BDS	private	1
1258	F	19	BDS	private	4
1259	F	20	BDS	private	2
1260	M	23	BDS	pubic	1
1261	M	23	BDS	private	4
1262	M	20	BDS	private	2
1263	F	20	BDS	private	2
1264	M	20	BDS	private	3
1265	F	21	BDS	public	4
1266	F	23	BDS	public	4
1267	F	19	BDS	public	1

1268	M	23	BDS	public	4
1269	F	20	BDS	Private	2
1270	F	21	BDS	Private	1
1271	M	19	BDS	Private	4
1272	F	19	BDS	Private	2
1273	F	18	BDS	public	2
1274	M	19	BDS	public	3
1275	M	20	BDS	public	4
1276	M	20	BDS	public	4
1277	F	21	BDS	private	1
1278	M	20	BDS	private	4
1279	F	23	BDS	private	2
1280	F	22	BDS	pubic	1
1281	F	22	BDS	private	4
1282	M	21	BDS	private	2
1283	F	20	BDS	private	2
1284	F	20	BDS	private	3
1285	M	21	BDS	public	4
1286	F	19	BDS	public	2
1287	F	19	BDS	public	1
1288	M	19	BDS	public	4
1289	M	20	BDS	Private	2
1290	M	23	BDS	Private	1
1291	F	23	BDS	Private	4
1292	M	20	BDS	Private	2
1293	F	20	BDS	public	2
1294	F	20	BDS	public	3
1295	F	21	BDS	public	4
1296	M	23	BDS	public	1
1297	F	19	BDS	private	4
1298	F	23	BDS	private	2
1299	M	20	BDS	private	1
1300	F	21	BDS	pubic	4
1301	F	19	BDS	private	2
1302	M	19	BDS	private	2
1303	M	18	BDS	private	3
1304	M	19	BDS	private	4
1305	F	20	BDS	public	4
1306	M	20	BDS	public	1
1307	F	21	BDS	public	4
1308	F	20	BDS	public	2
1309	F	23	BDS	Private	1
1310	M	22	BDS	Private	4
1311	F	22	BDS	Private	2
1312	F	21	BDS	Private	2
1313		20	BDS	public	3
1314	F	20	BDS	public	4

1315	M	21	BDS	public	4
1316	F	19	BDS	public	1
1317	F	19	BDS	private	4
1318	M	19	BDS	private	2
1319	F	20	BDS	private	1
1320	F	23	BDS	pubic	4
1321	M	23	BDS	private	2
1322	M	20	BDS	private	2
1323	M	20	BDS	private	3
1324	F	20	BDS	private	4
1325	M	21	BDS	public	2
1326	F	23	BDS	public	1
1327	F	19	BDS	public	4
1328	F	23	BDS	public	2
1329	M	20	BDS	Private	1
1330	F	21	BDS	Private	4
1331	F	19	BDS	Private	2
1332	M	19	BDS	Private	2
1333	F	18	BDS	public	3
1334	F	19	BDS	public	4
1335	M	20	BDS	public	1
1336	M	20	BDS	public	4
1337	M	21	BDS	private	2
1338	F	20	BDS	private	1
1339	M	23	BDS	private	4
1340	F	22	BDS	pubic	2
1341	F	22	BDS	private	2
1342	F	21	BDS	private	3
1343	M	20	BDS	private	4
1344	F	20	BDS	private	4
1345	F	21	BDS	public	1
1346	M	19	BDS	public	4
1347	F	19	BDS	public	2
1348	F	19	BDS	public	1
1349	M	20	BDS	Private	4
1350	M	23	BDS	Private	2
1351	M	23	BDS	Private	2
1352	F	20	BDS	Private	3
1353	M	20	BDS	public	4
1354	F	20	BDS	public	4
1355	F	21	BDS	public	1
1356	F	23	BDS	public	4
1357	M	19	BDS	private	2
1358	F	23	BDS	private	1
1359	F	20	BDS	private	4
1360	M	21	BDS	pubic	2
1361	F	19	BDS	private	2

1362	F	19	BDS	private	3
1363	M	18	BDS	private	4
1364	M	19	BDS	private	2
1365	M	20	BDS	public	1
1366	F	20	BDS	public	4
1367	M	21	BDS	public	2
1368	F	20	BDS	public	1
1369	F	23	BDS	Private	4
1370	F	22	BDS	Private	2
1371	M	22	BDS	Private	2
1372	F	21	BDS	Private	3
1373	F	20	BDS	public	4
1374	M	20	BDS	public	1
1375	F	21	BDS	public	4
1376	F	19	BDS	public	2
1377	M	19	BDS	private	1
1378	M	19	BDS	private	4
1379	M	20	BDS	private	2
1380	F	23	BDS	pubic	2
1381	M	23	BDS	private	3
1382	F	20	BDS	private	4
1383	F	20	BDS	private	4
1384	F	20	BDS	private	1
1385	M	21	BDS	public	4
1386	F	23	BDS	public	2
1387	F	19	BDS	public	1
1388	M	23	BDS	public	4
1389	F	20	BDS	Private	2
1390	F	21	BDS	Private	2
1391	M	19	BDS	Private	3
1392	M	19	BDS	Private	4
1393	M	18	BDS	public	4
1394	F	19	BDS	public	1
1395	M	20	BDS	public	4
1396	F	20	BDS	public	2
1397	F	21	BDS	private	1
1398	F	20	BDS	private	4
1399	M	23	BDS	private	2
1400	F	22	BDS	pubic	2
1401	F	22	BDS	private	3
1402	M	21	BDS	private	4
1403	F	20	BDS	private	2
1404	F	20	BDS	private	1
1405	M	21	BDS	public	4
1406	M	19	BDS	public	2
1407	M	19	BDS	public	1
1408	F	19	BDS	public	4

1409	M	20	BDS	Private	2
1410	F	23	BDS	Private	2
1411	F	23	BDS	Private	3
1412	F	20	BDS	Private	4
1413	M	20	BDS	public	1
1414	F	20	BDS	public	4
1415	F	21	BDS	public	2
1416	M	23	BDS	public	1
1417	F	19	BDS	private	4
1418	F	23	BDS	private	2
1419	M	20	BDS	private	2
1420	M	21	BDS	pubic	3
1421	M	19	BDS	private	4
1422	F	19	BDS	private	4
1423	M	18	BDS	private	1
1424	F	19	BDS	private	4
1425	F	20	BDS	public	2
1426	F	20	BDS	public	1
1427	M	21	BDS	public	4
1428	F	20	BDS	public	2
1429	F	23	BDS	Private	2
1430	M	22	BDS	Private	3
1431	M	22	BDS	Private	4
1432	M	21	BDS	Private	4
1433	M	20	BDS	public	1
1434	M	20	BDS	public	4
1435	M	21	BDS	public	2
1436	M	19	BDS	public	1
1437	M	19	BDS	private	4
1438	M	19	BDS	private	2
1439	M	20	BDS	private	2
1440	M	23	BDS	pubic	3
1441	M	23	BDS	private	4
1442	M	19	BDS	private	2
1443	M	20	BDS	private	1
1444	M	22	BDS	private	4
1445	M	22	BDS	public	2
1446	M	21	BDS	public	1
1447	M	20	BDS	public	4
1448	M	21	BDS	public	2
1449	M	19	BDS	Private	2
1450	F	19	BDS	Private	3
1451	F	18	BDS	Private	2
1452	F	19	BDS	Private	3
1453	F	20	BDS	public	4
1454	F	20	BDS	public	4
1455	F	21	BDS	public	1

1456	F	20	BDS	public	4
1457	F	23	BDS	private	2
1458	F	22	BDS	private	1
1459	F	22	BDS	private	4
1460	F	21	BDS	pubic	2
1461	F	20	BDS	private	2
1462	F	20	BDS	private	3
1463	F	21	BDS	private	4
1464	F	19	BDS	private	2
1465	F	19	BDS	public	1
1466	F	19	BDS	public	4
1467	F	20	BDS	public	2
1468	F	23	BDS	public	1
1469	F	23	BDS	private	4
1470	F	19	BDS	private	2
1471	F	20	BDS	private	2
1472	F	22	BDS	pubic	3
1473	F	22	BDS	private	3

Academic grade	Visual	Aural	Read/write	Kinesthetic	Student satisfaction
A	1	0	0	0	yes
B	1	0	0	0	no
B	0	1	0	0	no
C	0	1	0	0	no
D	1	0	0	0	no
D	0	1	0	0	yes
C	1	0	0	0	no
C	0	1	0	0	no
B	0	1	0	0	no
B	1	0	0	0	yes
B	0	1	0	0	no
B	0	1	0	0	no
B	1	0	0	0	yes
B	0	1	0	0	no
A	1	0	0	0	no
C	1	0	0	0	no
C	1	0	0	0	no
D	0	1	1	0	no
B	1	0	1	0	no
B	1	0	0	0	no
B	0	1	1	1	yes
D	1	1	1	1	yes
C	1	1	1	0	no
A	1	1	0	0	no
C	0	1	1	1	no
D	1	1	1	1	no
B	1	0	0	0	no
B	1	1	1	1	no
D	1	1	1	1	no
C	0	1	1	0	no
A	0	0	0	1	no
B	1	1	1	1	no
B	1	1	1	1	no
D	1	1	1	0	no
C	1	0	0	0	no
C	0	1	1	1	yes
D	0	1	1	0	no
C	0	0	1	0	no
C	1	0	0	1	no
D	0	1	1	1	no
D	1	1	1	1	no
C	1	1	1	0	yes
C	1	1	0	0	no
C	1	1	0	1	no
B	1	1	1	1	no

B	0	0	1	0	no
B	1	1	1	1	no
B	1	0	1	1	no
B	1	1	0	0	no
A	0	0	0	1	no
C	1	1	1	1	no
D	1	1	1	1	yes
C	0	0	0	1	no
D	1	1	0	1	no
C	0	0	1	0	no
B	1	1	0	0	no
D	1	1	1	1	no
C	1	0	0	1	yes
A	1	0	1	0	yes
D	1	0	1	0	no
C	0	1	0	1	no
C	1	1	1	1	no
C	1	1	1	1	no
B	0	0	1	0	no
B	1	1	1	1	no
D	1	1	1	1	no
C	1	0	0	1	no
D	0	0	0	1	yes
C	1	1	1	1	no
D	1	1	1	1	no
C	0	0	0	1	yes
B	1	1	1	1	yes
B	1	1	0	1	no
B	0	0	1	0	no
B	0	0	0	1	no
C	1	1	1	1	no
B	0	1	0	1	no
C	1	0	1	1	yes
B	0	0	0	1	no
C	1	0	0	1	no
D	1	1	1	1	no
C	0	0	0	1	no
B	0	1	0	1	yes
C	0	0	0	1	yes
D	1	1	0	1	no
C	0	0	0	1	no
B	1	0	0	1	no
D	1	0	1	1	no
B	0	0	0	1	no
C	1	1	0	1	yes
D	0	0	0	1	no
C	0	0	1	1	no

A	1	1	0	1	yes
C	0	0	0	1	no
A	1	0	1	1	yes
C	1	1	0	1	no
A	1	1	0	1	yes
B	1	0	0	1	no
A	0	0	1	1	no
A	0	0	0	1	yes
A	1	1	1	1	yes
B	1	0	0	0	no
A	1	0	0	0	no
A	0	1	0	0	no
A	0	1	0	0	no
B	1	0	0	0	yes
A	0	1	0	0	no
B	1	0	0	0	no
B	0	1	0	0	no
B	0	1	0	0	yes
D	1	0	0	0	no
C	0	1	0	0	no
C	0	1	0	0	yes
B	1	0	0	0	no
B	0	1	0	0	no
B	1	0	0	0	no
B	1	0	0	0	no
A	1	0	0	0	no
A	0	1	1	0	no
A	1	0	1	0	no
C	1	0	0	0	yes
D	0	1	1	1	yes
C	1	1	1	1	no
A	1	1	1	0	no
A	1	1	0	0	no
B	0	1	1	1	no
D	1	1	1	1	no
A	1	0	0	0	no
B	1	1	1	1	no
D	1	1	1	1	no
B	0	1	1	0	no
D	0	0	0	1	no
C	1	1	1	1	no
A	1	1	1	1	no
B	1	1	1	0	no
B	1	0	0	0	yes
C	0	1	1	1	no
D	0	1	1	0	no
D	0	0	1	0	no

C	1	0	0	1	no
C	0	1	1	1	no
B	1	1	1	1	yes
B	1	1	1	0	no
B	1	1	0	0	no
B	1	1	0	1	no
B	1	1	1	1	no
B	0	0	1	0	no
A	1	1	1	1	no
C	1	0	1	1	no
C	1	1	0	0	no
D	0	0	0	1	no
B	1	1	1	1	yes
B	1	1	1	1	no
B	0	0	0	1	no
D	1	1	0	1	no
C	0	0	1	0	no
A	1	1	0	0	no
C	1	1	1	1	yes
D	1	0	0	1	yes
B	1	0	1	0	no
B	1	0	1	0	no
D	0	1	0	1	no
C	1	1	1	1	no
A	1	1	1	1	no
B	0	0	1	0	no
B	1	1	1	1	no
D	1	1	1	1	no
C	1	0	0	1	yes
C	0	0	0	1	no
D	1	1	1	1	no
C	1	1	1	1	yes
C	0	0	0	1	yes
D	1	1	1	1	no
D	1	1	0	1	no
C	0	0	1	0	no
C	0	0	0	1	no
C	1	1	1	1	no
B	0	1	0	1	yes
B	1	0	1	1	no
B	0	0	0	1	no
B	1	0	0	1	no
B	1	1	1	1	no
A	0	0	0	1	yes
C	0	1	0	1	yes
D	0	0	0	1	no
C	1	1	0	1	no

D	0	0	0	1	no
C	1	0	0	1	no
B	1	0	1	1	no
D	0	0	0	1	yes
C	1	1	0	1	no
A	0	0	0	1	no
D	0	0	1	1	yes
C	1	1	0	1	no
C	0	0	0	1	yes
C	1	0	1	1	no
B	1	1	0	1	yes
B	1	1	0	1	no
D	1	0	0	1	no
C	0	0	1	1	yes
D	0	0	0	1	yes
C	1	1	1	1	no
D	1	0	0	0	no
C	1	0	0	0	no
B	0	1	0	0	no
B	0	1	0	0	yes
B	1	0	0	0	no
B	0	1	0	0	no
C	1	0	0	0	no
B	0	1	0	0	yes
C	0	1	0	0	no
B	1	0	0	0	no
C	0	1	0	0	yes
D	0	1	0	0	no
C	1	0	0	0	no
B	0	1	0	0	no
C	1	0	0	0	no
D	1	0	0	0	no
C	1	0	0	0	no
B	0	1	1	0	no
D	1	0	1	0	yes
B	1	0	0	0	yes
C	0	1	1	1	no
D	1	1	1	1	no
C	1	1	1	0	no
A	1	1	0	0	no
C	0	1	1	1	no
A	1	1	1	1	no
C	1	0	0	0	no
A	1	1	1	1	no
B	1	1	1	1	no
A	0	1	1	0	no
A	0	0	0	1	no

A	1	1	1	1	no
B	1	1	1	1	no
A	1	1	1	0	yes
A	1	0	0	0	no
A	0	1	1	1	no
B	0	1	1	0	no
A	0	0	1	0	no
B	1	0	0	1	no
B	0	1	1	1	yes
B	1	1	1	1	no
D	1	1	1	0	no
C	1	1	0	0	no
C	1	1	0	1	no
B	1	1	1	1	no
B	0	0	1	0	no
B	1	1	1	1	no
B	1	0	1	1	no
A	1	1	0	0	no
A	0	0	0	1	yes
A	1	1	1	1	no
C	1	1	1	1	no
D	0	0	0	1	no
C	1	1	0	1	no
A	0	0	1	0	no
A	1	1	0	0	yes
B	1	1	1	1	yes
D	1	0	0	1	no
A	1	0	1	0	no
B	1	0	1	0	no
D	0	1	0	1	no
B	1	1	1	1	no
D	1	1	1	1	no
C	0	0	1	0	no
A	1	1	1	1	no
B	1	1	1	1	yes
B	1	0	0	1	no
C	0	0	0	1	no
D	1	1	1	1	yes
D	1	1	1	1	yes
C	0	0	0	1	no
C	1	1	1	1	no
B	1	1	0	1	no
B	0	0	1	0	no
B	0	0	0	1	no
B	1	1	1	1	yes
B	0	1	0	1	no
B	1	0	1	1	no

A	0	0	0	1	no
C	1	0	0	1	no
C	1	1	1	1	yes
D	0	0	0	1	yes
B	0	1	0	1	no
B	0	0	0	1	no
B	1	1	0	1	no
D	0	0	0	1	no
C	1	0	0	1	no
A	1	0	1	1	yes
C	0	0	0	1	no
D	1	1	0	1	no
B	0	0	0	1	yes
B	0	0	1	1	no
D	1	1	0	1	yes
C	0	0	0	1	no
A	1	0	1	1	yes
B	1	1	0	1	no
B	1	1	0	1	no
D	1	0	0	1	yes
C	0	0	1	1	yes
C	0	0	0	1	no
D	1	1	1	1	no
C	1	0	0	0	no
C	1	0	0	0	no
D	0	1	0	0	yes
D	0	1	0	0	no
C	1	0	0	0	no
C	0	1	0	0	no
C	1	0	0	0	yes
B	0	1	0	0	no
B	0	1	0	0	no
B	1	0	0	0	yes
B	0	1	0	0	no
B	0	1	0	0	no
A	1	0	0	0	no
C	0	1	0	0	no
D	1	0	0	0	no
C	1	0	0	0	no
D	1	0	0	0	no
C	0	1	1	0	yes
B	1	0	1	0	yes
D	1	0	0	0	no
C	0	1	1	1	no
A	1	1	1	1	no
D	1	1	1	0	no
C	1	1	0	0	no

C	0	1	1	1	no
C	1	1	1	1	no
B	1	0	0	0	no
B	1	1	1	1	no
D	1	1	1	1	no
C	0	1	1	0	no
D	0	0	0	1	no
C	1	1	1	1	no
D	1	1	1	1	yes
C	1	1	1	0	no
B	1	0	0	0	no
B	0	1	1	1	no
B	0	1	1	0	no
B	0	0	1	0	no
C	1	0	0	1	yes
B	0	1	1	1	no
C	1	1	1	1	no
B	1	1	1	0	no
C	1	1	0	0	no
D	1	1	0	1	no
C	1	1	1	1	no
B	0	0	1	0	no
C	1	1	1	1	no
D	1	0	1	1	no
C	1	1	0	0	yes
B	0	0	0	1	no
D	1	1	1	1	no
B	1	1	1	1	no
C	0	0	0	1	no
D	1	1	0	1	no
C	0	0	1	0	yes
A	1	1	0	0	yes
C	1	1	1	1	no
A	1	0	0	1	no
C	1	0	1	0	no
A	1	0	1	0	no
B	0	1	0	1	no
A	1	1	1	1	no
A	1	1	1	1	no
A	0	0	1	0	no
B	1	1	1	1	yes
A	1	1	1	1	no
A	1	0	0	1	no
A	0	0	0	1	yes
B	1	1	1	1	yes
A	1	1	1	1	no
B	0	0	0	1	no

B	1	1	1	1	no
B	1	1	0	1	no
D	0	0	1	0	no
C	0	0	0	1	yes
C	1	1	1	1	no
B	0	1	0	1	no
B	1	0	1	1	no
B	0	0	0	1	no
B	1	0	0	1	yes
A	1	1	1	1	yes
A	0	0	0	1	no
A	0	1	0	1	no
C	0	0	0	1	no
D	1	1	0	1	no
C	0	0	0	1	no
A	1	0	0	1	yes
A	1	0	1	1	no
B	0	0	0	1	no
D	1	1	0	1	yes
A	0	0	0	1	no
B	0	0	1	1	yes
D	1	1	0	1	no
B	0	0	0	1	yes
D	1	0	1	1	no
C	1	1	0	1	no
A	1	1	0	1	yes
B	1	0	0	1	yes
B	0	0	1	1	no
C	0	0	0	1	no
D	1	1	1	1	no
D	1	0	0	0	no
C	1	0	0	0	yes
C	0	1	0	0	no
B	0	1	0	0	no
B	1	0	0	0	no
B	0	1	0	0	yes
B	1	0	0	0	no
B	0	1	0	0	no
B	0	1	0	0	yes
A	1	0	0	0	no
C	0	1	0	0	no
C	0	1	0	0	no
D	1	0	0	0	no
B	0	1	0	0	no
B	1	0	0	0	no
B	1	0	0	0	no
D	1	0	0	0	yes

C	0	1	1	0	yes
A	1	0	1	0	no
C	1	0	0	0	no
D	0	1	1	1	no
B	1	1	1	1	no
B	1	1	1	0	no
D	1	1	0	0	no
C	0	1	1	1	no
A	1	1	1	1	no
B	1	0	0	0	no
B	1	1	1	1	no
D	1	1	1	1	no
C	0	1	1	0	no
C	0	0	0	1	no
D	1	1	1	1	yes
C	1	1	1	1	no
C	1	1	1	0	no
D	1	0	0	0	no
D	0	1	1	1	no
C	0	1	1	0	no
C	0	0	1	0	yes
C	1	0	0	1	no
B	0	1	1	1	no
B	1	1	1	1	no
B	1	1	1	0	no
B	1	1	0	0	no
B	1	1	0	1	no
A	1	1	1	1	no
C	0	0	1	0	no
D	1	1	1	1	no
C	1	0	1	1	yes
D	1	1	0	0	no
C	0	0	0	1	no
B	1	1	1	1	no
D	1	1	1	1	no
C	0	0	0	1	no
A	1	1	0	1	yes
D	0	0	1	0	yes
C	1	1	0	0	no
C	1	1	1	1	no
C	1	0	0	1	no
B	1	0	1	0	no
B	1	0	1	0	no
D	0	1	0	1	no
C	1	1	1	1	no
D	1	1	1	1	no
C	0	0	1	0	yes

D	1	1	1	1	no
C	1	1	1	1	no
B	1	0	0	1	yes
B	0	0	0	1	yes
B	1	1	1	1	no
B	1	1	1	1	no
C	0	0	0	1	no
B	1	1	1	1	no
C	1	1	0	1	no
B	0	0	1	0	yes
C	0	0	0	1	no
D	1	1	1	1	no
C	0	1	0	1	no
B	1	0	1	1	no
C	0	0	0	1	yes
D	1	0	0	1	yes
C	1	1	1	1	no
B	0	0	0	1	no
D	0	1	0	1	no
B	0	0	0	1	no
C	1	1	0	1	no
D	0	0	0	1	yes
C	1	0	0	1	no
A	1	0	1	1	no
C	0	0	0	1	yes
A	1	1	0	1	no
C	0	0	0	1	yes
A	0	0	1	1	no
B	1	1	0	1	yes
A	0	0	0	1	no
A	1	0	1	1	no
A	1	1	0	1	yes
B	1	1	0	1	yes
A	1	0	0	1	no
A	0	0	1	1	no
A	0	0	0	1	no
B	1	1	1	1	no
A	1	0	0	0	yes
B	1	0	0	0	no
B	0	1	0	0	no
B	0	1	0	0	no
D	1	0	0	0	yes
C	0	1	0	0	no
C	1	0	0	0	no
B	0	1	0	0	yes
B	0	1	0	0	no
B	1	0	0	0	no

B	0	1	0	0	no
A	0	1	0	0	no
A	1	0	0	0	no
A	0	1	0	0	no
C	1	0	0	0	no
D	1	0	0	0	yes
C	1	0	0	0	yes
A	0	1	1	0	no
A	1	0	1	0	no
B	1	0	0	0	no
D	0	1	1	1	no
A	1	1	1	1	no
B	1	1	1	0	no
D	1	1	0	0	no
B	0	1	1	1	no
D	1	1	1	1	no
C	1	0	0	0	no
A	1	1	1	1	no
B	1	1	1	1	no
B	0	1	1	0	no
C	0	0	0	1	yes
D	1	1	1	1	no
D	1	1	1	1	no
C	1	1	1	0	no
C	1	0	0	0	no
B	0	1	1	1	no
B	0	1	1	0	yes
B	0	0	1	0	no
B	1	0	0	1	no
B	0	1	1	1	no
B	1	1	1	1	no
A	1	1	1	0	no
C	1	1	0	0	no
C	1	1	0	1	no
D	1	1	1	1	no
B	0	0	1	0	no
B	1	1	1	1	yes
B	1	0	1	1	no
D	1	1	0	0	no
C	0	0	0	1	no
A	1	1	1	1	no
C	1	1	1	1	no
D	0	0	0	1	yes
B	1	1	0	1	yes
B	0	0	1	0	no
D	1	1	0	0	no
C	1	1	1	1	no

A	1	0	0	1	no
B	1	0	1	0	no
B	1	0	1	0	no
D	0	1	0	1	no
C	1	1	1	1	no
C	1	1	1	1	yes
D	0	0	1	0	no
C	1	1	1	1	no
C	1	1	1	1	yes
D	1	0	0	1	yes
D	0	0	0	1	no
C	1	1	1	1	no
C	1	1	1	1	no
C	0	0	0	1	no
B	1	1	1	1	no
B	1	1	0	1	yes
B	0	0	1	0	no
B	0	0	0	1	no
B	1	1	1	1	no
A	0	1	0	1	no
C	1	0	1	1	yes
D	0	0	0	1	yes
C	1	0	0	1	no
D	1	1	1	1	no
C	0	0	0	1	no
B	0	1	0	1	no
D	0	0	0	1	no
C	1	1	0	1	yes
A	0	0	0	1	no
D	1	0	0	1	no
C	1	0	1	1	yes
C	0	0	0	1	no
C	1	1	0	1	yes
B	0	0	0	1	no
B	0	0	1	1	yes
D	1	1	0	1	no
C	0	0	0	1	no
D	1	0	1	1	yes
C	1	1	0	1	yes
D	1	1	0	1	no
C	1	0	0	1	no
B	0	0	1	1	no
B	0	0	0	1	no
B	1	1	1	1	yes
B	1	0	0	0	no
C	1	0	0	0	no
B	0	1	0	0	no

C	0	1	0	0	yes
B	1	0	0	0	no
C	0	1	0	0	no
D	1	0	0	0	yes
C	0	1	0	0	no
B	0	1	0	0	no
C	1	0	0	0	no
D	0	1	0	0	no
C	0	1	0	0	no
B	1	0	0	0	no
D	0	1	0	0	no
B	1	0	0	0	yes
C	1	0	0	0	yes
D	1	0	0	0	no
C	0	1	1	0	no
A	1	0	1	0	no
C	1	0	0	0	no
A	0	1	1	1	no
C	1	1	1	1	no
A	1	1	1	0	no
B	1	1	0	0	no
A	0	1	1	1	no
A	1	1	1	1	no
A	1	0	0	0	no
B	1	1	1	1	no
A	1	1	1	1	no
A	0	1	1	0	yes
A	0	0	0	1	no
B	1	1	1	1	no
A	1	1	1	1	no
B	1	1	1	0	no
B	1	0	0	0	no
B	0	1	1	1	yes
D	0	1	1	0	no
C	0	0	1	0	no
C	1	0	0	1	no
B	0	1	1	1	no
B	1	1	1	1	no
B	1	1	1	0	no
B	1	1	0	0	no
A	1	1	0	1	no
A	1	1	1	1	no
A	0	0	1	0	yes
C	1	1	1	1	no
D	1	0	1	1	no
C	1	1	0	0	no
A	0	0	0	1	no

A	1	1	1	1	no
B	1	1	1	1	yes
D	0	0	0	1	yes
A	1	1	0	1	no
B	0	0	1	0	no
D	1	1	0	0	no
B	1	1	1	1	no
D	1	0	0	1	no
C	1	0	1	0	no
A	1	0	1	0	no
B	0	1	0	1	no
B	1	1	1	1	yes
C	1	1	1	1	no
D	0	0	1	0	no
D	1	1	1	1	yes
C	1	1	1	1	yes
C	1	0	0	1	no
B	0	0	0	1	no
B	1	1	1	1	no
B	1	1	1	1	no
B	0	0	0	1	no
B	1	1	1	1	yes
B	1	1	0	1	no
A	0	0	1	0	no
C	0	0	0	1	no
C	1	1	1	1	no
D	0	1	0	1	yes
B	1	0	1	1	yes
B	0	0	0	1	no
B	1	0	0	1	no
D	1	1	1	1	no
C	0	0	0	1	no
A	0	1	0	1	no
C	0	0	0	1	yes
D	1	1	0	1	no
B	0	0	0	1	no
B	1	0	0	1	yes
D	1	0	1	1	no
C	0	0	0	1	yes
A	1	1	0	1	no
B	0	0	0	1	yes
B	0	0	1	1	no
D	1	1	0	1	no
C	0	0	0	1	yes
C	1	0	1	1	yes
D	1	1	0	1	no
C	1	1	0	1	no

C	1	0	0	1	no
D	0	0	1	1	no
D	0	0	0	1	yes
C	1	1	1	1	no
C	1	0	0	0	no
C	1	0	0	0	no
B	0	1	0	0	yes
B	0	1	0	0	no
B	1	0	0	0	no
B	0	1	0	0	yes
B	1	0	0	0	no
A	0	1	0	0	no
C	0	1	0	0	no
D	1	0	0	0	no
C	0	1	0	0	no
D	0	1	0	0	no
C	1	0	0	0	no
B	0	1	0	0	yes
D	1	0	0	0	yes
C	1	0	0	0	no
A	1	0	0	0	no
D	0	1	1	0	no
C	1	0	1	0	no
C	1	0	0	0	no
C	0	1	1	1	no
B	1	1	1	1	no
B	1	1	1	0	no
D	1	1	0	0	no
C	0	1	1	1	no
D	1	1	1	1	no
C	1	0	0	0	no
D	1	1	1	1	no
C	1	1	1	1	yes
B	0	1	1	0	no
B	0	0	0	1	no
B	1	1	1	1	no
B	1	1	1	1	no
C	1	1	1	0	no
B	1	0	0	0	yes
C	0	1	1	1	no
B	0	1	1	0	no
C	0	0	1	0	no
D	1	0	0	1	no
C	0	1	1	1	no
B	1	1	1	1	no
C	1	1	1	0	no
D	1	1	0	0	no

C	1	1	0	1	no
B	1	1	1	1	yes
D	0	0	1	0	no
B	1	1	1	1	no
C	1	0	1	1	no
D	1	1	0	0	no
C	0	0	0	1	no
A	1	1	1	1	yes
C	1	1	1	1	yes
A	0	0	0	1	no
C	1	1	0	1	no
A	0	0	1	0	no
B	1	1	0	0	no
A	1	1	1	1	no
A	1	0	0	1	no
A	1	0	1	0	no
B	1	0	1	0	no
A	0	1	0	1	yes
A	1	1	1	1	no
A	1	1	1	1	no
B	0	0	1	0	yes
A	1	1	1	1	yes
B	1	1	1	1	no
B	1	0	0	1	no
B	0	0	0	1	no
D	1	1	1	1	no
C	1	1	1	1	no
C	0	0	0	1	yes
B	1	1	1	1	no
B	1	1	0	1	no
B	0	0	1	0	no
B	0	0	0	1	no
A	1	1	1	1	yes
A	0	1	0	1	yes
A	1	0	1	1	no
C	0	0	0	1	no
D	1	0	0	1	no
C	1	1	1	1	no
A	0	0	0	1	no
A	0	1	0	1	yes
B	0	0	0	1	no
D	1	1	0	1	no
A	0	0	0	1	yes
B	1	0	0	1	no
D	1	0	1	1	yes
B	0	0	0	1	no
D	1	1	0	1	yes

C	0	0	0	1	no
A	0	0	1	1	no
B	1	1	0	1	yes
B	0	0	0	1	yes
C	1	0	1	1	no
D	1	1	0	1	no
D	1	1	0	1	no
C	1	0	0	1	no
C	0	0	1	1	yes
B	0	0	0	1	no
B	1	1	1	1	no
B	1	0	0	0	no
B	1	0	0	0	yes
B	0	1	0	0	no
B	0	1	0	0	no
A	1	0	0	0	yes
C	0	1	0	0	no
C	1	0	0	0	no
D	0	1	0	0	no
B	0	1	0	0	no
B	1	0	0	0	no
B	0	1	0	0	no
D	0	1	0	0	no
C	1	0	0	0	yes
A	0	1	0	0	yes
C	1	0	0	0	no
D	1	0	0	0	no
B	1	0	0	0	no
B	0	1	1	0	no
D	1	0	1	0	no
C	1	0	0	0	no
A	0	1	1	1	no
B	1	1	1	1	no
B	1	1	1	0	no
D	1	1	0	0	no
C	0	1	1	1	no
C	1	1	1	1	no
D	1	0	0	0	no
C	1	1	1	1	yes
C	1	1	1	1	no
D	0	1	1	0	no
D	0	0	0	1	no
C	1	1	1	1	no
C	1	1	1	1	no
C	1	1	1	0	yes
B	1	0	0	0	no
B	0	1	1	1	no

B	0	1	1	0	no
B	0	0	1	0	no
B	1	0	0	1	no
A	0	1	1	1	no
C	1	1	1	1	no
D	1	1	1	0	no
C	1	1	0	0	no
D	1	1	0	1	yes
C	1	1	1	1	no
B	0	0	1	0	no
D	1	1	1	1	no
C	1	0	1	1	no
A	1	1	0	0	no
D	0	0	0	1	yes
C	1	1	1	1	yes
C	1	1	1	1	no
C	0	0	0	1	no
B	1	1	0	1	no
B	0	0	1	0	no
D	1	1	0	0	no
C	1	1	1	1	no
D	1	0	0	1	no
C	1	0	1	0	no
D	1	0	1	0	yes
C	0	1	0	1	no
B	1	1	1	1	no
B	1	1	1	1	yes
B	0	0	1	0	yes
B	1	1	1	1	no
C	1	1	1	1	no
B	1	0	0	1	no
C	0	0	0	1	no
B	1	1	1	1	no
C	1	1	1	1	yes
D	0	0	0	1	no
C	1	1	1	1	no
B	1	1	0	1	no
C	0	0	1	0	no
D	0	0	0	1	yes
C	1	1	1	1	yes
B	0	1	0	1	no
D	1	0	1	1	no
B	0	0	0	1	no
C	1	0	0	1	no
D	1	1	1	1	no
C	0	0	0	1	yes
A	0	1	0	1	no

C	0	0	0	1	no
A	1	1	0	1	yes
C	0	0	0	1	no
A	1	0	0	1	yes
B	1	0	1	1	no
A	0	0	0	1	yes
A	1	1	0	1	no
A	0	0	0	1	no
B	0	0	1	1	yes
A	1	1	0	1	yes
A	0	0	0	1	no
A	1	0	1	1	no
B	1	1	0	1	no
A	1	1	0	1	no
B	1	0	0	1	yes
B	0	0	1	1	no
B	0	0	0	1	no
D	1	1	1	1	no
C	1	0	0	0	yes
C	1	0	0	0	no
B	0	1	0	0	no
B	0	1	0	0	yes
B	1	0	0	0	no
B	0	1	0	0	no
A	1	0	0	0	no
A	0	1	0	0	no
A	0	1	0	0	no
C	1	0	0	0	no
D	0	1	0	0	no
C	0	1	0	0	yes
A	1	0	0	0	yes
A	0	1	0	0	no
B	1	0	0	0	no
D	1	0	0	0	no
A	1	0	0	0	no
B	0	1	1	0	no
D	1	0	1	0	no
B	1	0	0	0	no
D	0	1	1	1	no
C	1	1	1	1	no
A	1	1	1	0	no
B	1	1	0	0	no
B	0	1	1	1	no
C	1	1	1	1	no
D	1	0	0	0	yes
D	1	1	1	1	no
C	1	1	1	1	no

C	0	1	1	0	no
B	0	0	0	1	no
B	1	1	1	1	no
B	1	1	1	1	yes
B	1	1	1	0	no
B	1	0	0	0	no
B	0	1	1	1	no
A	0	1	1	0	no
C	0	0	1	0	no
C	1	0	0	1	no
D	0	1	1	1	no
B	1	1	1	1	no
B	1	1	1	0	no
B	1	1	0	0	yes
D	1	1	0	1	no
C	1	1	1	1	no
A	0	0	1	0	no
C	1	1	1	1	no
D	1	0	1	1	no
B	1	1	0	0	yes
B	0	0	0	1	yes
D	1	1	1	1	no
C	1	1	1	1	no
A	0	0	0	1	no
B	1	1	0	1	no
B	0	0	1	0	no
D	1	1	0	0	no
C	1	1	1	1	no
C	1	0	0	1	no
D	1	0	1	0	yes
C	1	0	1	0	no
C	0	1	0	1	no
D	1	1	1	1	yes
D	1	1	1	1	yes
C	0	0	1	0	no
C	1	1	1	1	no
C	1	1	1	1	no
B	1	0	0	1	no
B	0	0	0	1	no
B	1	1	1	1	yes
B	1	1	1	1	no
B	0	0	0	1	no
A	1	1	1	1	no
C	1	1	0	1	no
D	0	0	1	0	yes
C	0	0	0	1	yes
D	1	1	1	1	no

C	0	1	0	1	no
B	1	0	1	1	no
D	0	0	0	1	no
C	1	0	0	1	no
A	1	1	1	1	yes
D	0	0	0	1	no
C	0	1	0	1	no
C	0	0	0	1	yes
C	1	1	0	1	no
B	0	0	0	1	yes
B	1	0	0	1	no
D	1	0	1	1	yes
C	0	0	0	1	no
D	1	1	0	1	no
C	0	0	0	1	yes
D	0	0	1	1	yes
C	1	1	0	1	no
B	0	0	0	1	no
B	1	0	1	1	no
B	1	1	0	1	no
B	1	1	0	1	yes
C	1	0	0	1	no
B	0	0	1	1	no
C	0	0	0	1	no
B	1	1	1	1	yes
C	1	0	0	0	no
D	1	0	0	0	no
C	0	1	0	0	yes
B	0	1	0	0	no
C	1	0	0	0	no
D	0	1	0	0	no
C	1	0	0	0	no
B	0	1	0	0	no
D	0	1	0	0	no
B	1	0	0	0	no
C	0	1	0	0	yes
D	0	1	0	0	yes
C	1	0	0	0	no
A	0	1	0	0	no
C	1	0	0	0	no
A	1	0	0	0	no
C	1	0	0	0	no
A	0	1	1	0	no
B	1	0	1	0	no
A	1	0	0	0	no
A	0	1	1	1	no
A	1	1	1	1	no

B	1	1	1	0	no
A	1	1	0	0	no
A	0	1	1	1	no
A	1	1	1	1	yes
B	1	0	0	0	no
A	1	1	1	1	no
B	1	1	1	1	no
B	0	1	1	0	no
B	0	0	0	1	no
D	1	1	1	1	yes
C	1	1	1	1	no
C	1	1	1	0	no
B	1	0	0	0	no
B	0	1	1	1	no
B	0	1	1	0	no
B	0	0	1	0	no
A	1	0	0	1	no
A	0	1	1	1	no
A	1	1	1	1	no
C	1	1	1	0	yes
D	1	1	0	0	no
C	1	1	0	1	no
A	1	1	1	1	no
A	0	0	1	0	no
B	1	1	1	1	no
D	1	0	1	1	yes
A	1	1	0	0	yes
B	0	0	0	1	no
D	1	1	1	1	no
B	1	1	1	1	no
D	0	0	0	1	no
C	1	1	0	1	no
A	0	0	1	0	no
B	1	1	0	0	no
B	1	1	1	1	no
C	1	0	0	1	yes
D	1	0	1	0	no
D	1	0	1	0	no
C	0	1	0	1	yes
C	1	1	1	1	yes
B	1	1	1	1	no
B	0	0	1	0	no
B	1	1	1	1	no
B	1	1	1	1	no
B	1	0	0	1	no
B	0	0	0	1	yes
A	1	1	1	1	no

C	1	1	1	1	no
C	0	0	0	1	no
D	1	1	1	1	no
B	1	1	0	1	yes
B	0	0	1	0	yes
B	0	0	0	1	no
D	1	1	1	1	no
C	0	1	0	1	no
A	1	0	1	1	no
C	0	0	0	1	no
D	1	0	0	1	yes
B	1	1	1	1	no
B	0	0	0	1	no
D	0	1	0	1	yes
C	0	0	0	1	no
A	1	1	0	1	yes
B	0	0	0	1	no
B	1	0	0	1	yes
D	1	0	1	1	no
C	0	0	0	1	no
C	1	1	0	1	yes
D	0	0	0	1	yes
C	0	0	1	1	no
C	1	1	0	1	no
D	0	0	0	1	no
D	1	0	1	1	no
C	1	1	0	1	yes
C	1	1	0	1	no
C	1	0	0	1	no
B	0	0	1	1	no
B	0	0	0	1	yes
B	1	1	1	1	no
B	1	0	0	0	no
B	1	0	0	0	yes
A	0	1	0	0	no
C	0	1	0	0	no
D	1	0	0	0	no
C	0	1	0	0	no
D	1	0	0	0	no
C	0	1	0	0	no
B	0	1	0	0	no
D	1	0	0	0	yes
C	0	1	0	0	yes
A	0	1	0	0	no
D	1	0	0	0	no
C	0	1	0	0	no
C	1	0	0	0	no

C	1	0	0	0	no
B	1	0	0	0	no
B	0	1	1	0	no
D	1	0	1	0	no
C	1	0	0	0	no
D	0	1	1	1	no
C	1	1	1	1	no
D	1	1	1	0	no
C	1	1	0	0	no
B	0	1	1	1	yes
B	1	1	1	1	no
B	1	0	0	0	no
B	1	1	1	1	no
C	1	1	1	1	no
B	0	1	1	0	no
C	0	0	0	1	yes
B	1	1	1	1	no
C	1	1	1	1	no
D	1	1	1	0	no
C	1	0	0	0	no
B	0	1	1	1	no
C	0	1	1	0	no
D	0	0	1	0	no
C	1	0	0	1	no
B	0	1	1	1	no
D	1	1	1	1	yes
B	1	1	1	0	no
C	1	1	0	0	no
D	1	1	0	1	no
C	1	1	1	1	no
A	0	0	1	0	no
C	1	1	1	1	yes
A	1	0	1	1	yes
C	1	1	0	0	no
A	0	0	0	1	no
B	1	1	1	1	no
A	1	1	1	1	no
A	0	0	0	1	no
A	1	1	0	1	no
B	0	0	1	0	no
A	1	1	0	0	no
A	1	1	1	1	yes
A	1	0	0	1	no
B	1	0	1	0	no
A	1	0	1	0	yes
B	0	1	0	1	yes
B	1	1	1	1	no

B	1	1	1	1	no
D	0	0	1	0	no
C	1	1	1	1	no
C	1	1	1	1	no
B	1	0	0	1	yes
B	0	0	0	1	no
B	1	1	1	1	no
B	1	1	1	1	no
A	0	0	0	1	no
A	1	1	1	1	yes
A	1	1	0	1	yes
C	0	0	1	0	no
D	0	0	0	1	no
C	1	1	1	1	no
A	0	1	0	1	no
A	1	0	1	1	no
B	0	0	0	1	yes
D	1	0	0	1	no
A	1	1	1	1	no
B	0	0	0	1	yes
D	0	1	0	1	no
B	0	0	0	1	yes
D	1	1	0	1	no
C	0	0	0	1	yes
A	1	0	0	1	no
B	1	0	1	1	no
B	0	0	0	1	yes
C	1	1	0	1	yes
D	0	0	0	1	no
D	0	0	1	1	no
C	1	1	0	1	no
C	0	0	0	1	no
B	1	0	1	1	yes
B	1	1	0	1	no
B	1	1	0	1	no
B	1	0	0	1	no
B	0	0	1	1	yes
B	0	0	0	1	no
A	1	1	1	1	no
C	1	0	0	0	yes
C	1	0	0	0	no
D	0	1	0	0	no
B	0	1	0	0	no
B	1	0	0	0	no
B	0	1	0	0	no
D	1	0	0	0	no
C	0	1	0	0	no

A	0	1	0	0	yes
C	1	0	0	0	yes
D	0	1	0	0	no
B	0	1	0	0	no
B	1	0	0	0	no
D	0	1	0	0	no
C	1	0	0	0	no
A	1	0	0	0	no
B	1	0	0	0	no
B	0	1	1	0	no
D	1	0	1	0	no
C	1	0	0	0	no
C	0	1	1	1	no
D	1	1	1	1	no
C	1	1	1	0	no
C	1	1	0	0	yes
D	0	1	1	1	no
D	1	1	1	1	no
C	1	0	0	0	no
C	1	1	1	1	no
C	1	1	1	1	no
B	0	1	1	0	yes
B	0	0	0	1	no
B	1	1	1	1	no
B	1	1	1	1	no
B	1	1	1	0	no
A	1	0	0	0	no
C	0	1	1	1	no
D	0	1	1	0	no
C	0	0	1	0	no
D	1	0	0	1	no
C	0	1	1	1	yes
B	1	1	1	1	no
D	1	1	1	0	no
C	1	1	0	0	no
A	1	1	0	1	no
D	1	1	1	1	no
C	0	0	1	0	yes
C	1	1	1	1	yes
C	1	0	1	1	no
B	1	1	0	0	no
B	0	0	0	1	no
D	1	1	1	1	no
C	1	1	1	1	no
D	0	0	0	1	no
C	1	1	0	1	no
D	0	0	1	0	no

C	1	1	0	0	yes
B	1	1	1	1	no
B	1	0	0	1	no
B	1	0	1	0	yes
B	1	0	1	0	yes
C	0	1	0	1	no
B	1	1	1	1	no
C	1	1	1	1	no
B	0	0	1	0	no
C	1	1	1	1	no
D	1	1	1	1	yes
C	1	0	0	1	no
B	0	0	0	1	no
C	1	1	1	1	no
D	1	1	1	1	no
C	0	0	0	1	yes
B	1	1	1	1	yes
D	1	1	0	1	no
B	0	0	1	0	no
C	0	0	0	1	no
D	1	1	1	1	no
C	0	1	0	1	no
A	1	0	1	1	yes
C	0	0	0	1	no
A	1	0	0	1	no
C	1	1	1	1	yes
A	0	0	0	1	no
B	0	1	0	1	yes
A	0	0	0	1	no
A	1	1	0	1	yes
A	0	0	0	1	no
B	1	0	0	1	no
A	1	0	1	1	yes
A	0	0	0	1	yes
A	1	1	0	1	no
B	0	0	0	1	no
A	0	0	1	1	no
B	1	1	0	1	no
B	0	0	0	1	yes
B	1	0	1	1	no
D	1	1	0	1	no
C	1	1	0	1	no
C	1	0	0	1	yes
B	0	0	1	1	no
B	0	0	0	1	no
B	1	1	1	1	yes
B	1	0	0	0	no

A	1	0	0	0	no
A	0	1	0	0	no
A	0	1	0	0	no
C	1	0	0	0	no
D	0	1	0	0	no
C	1	0	0	0	no
A	0	1	0	0	yes
A	0	1	0	0	yes
B	1	0	0	0	no
D	0	1	0	0	no
A	0	1	0	0	no
B	1	0	0	0	no
D	0	1	0	0	no
B	1	0	0	0	no
D	1	0	0	0	no
C	1	0	0	0	no
A	0	1	1	0	no
B	1	0	1	0	no
B	1	0	0	0	no
C	0	1	1	1	no
D	1	1	1	1	no
D	1	1	1	0	yes
C	1	1	0	0	no
C	0	1	1	1	no
B	1	1	1	1	no
B	1	0	0	0	no
B	1	1	1	1	no
B	1	1	1	1	yes
B	0	1	1	0	no
B	0	0	0	1	no
A	1	1	1	1	no
C	1	1	1	1	no
C	1	1	1	0	no
D	1	0	0	0	no
B	0	1	1	1	no
B	0	1	1	0	no
B	0	0	1	0	no
D	1	0	0	1	yes
C	0	1	1	1	no
A	1	1	1	1	no
C	1	1	1	0	no
D	1	1	0	0	no
B	1	1	0	1	no
B	1	1	1	1	yes
D	0	0	1	0	yes
C	1	1	1	1	no
A	1	0	1	1	no

B	1	1	0	0	no
B	0	0	0	1	no
D	1	1	1	1	no
C	1	1	1	1	no
C	0	0	0	1	no
D	1	1	0	1	no
C	0	0	1	0	yes
C	1	1	0	0	no
D	1	1	1	1	no
D	1	0	0	1	yes
C	1	0	1	0	yes
C	1	0	1	0	no
C	0	1	0	1	no
B	1	1	1	1	no
B	1	1	1	1	no
B	0	0	1	0	no
B	1	1	1	1	yes
B	1	1	1	1	no
A	1	0	0	1	no
C	0	0	0	1	no
D	1	1	1	1	no
C	1	1	1	1	yes
D	0	0	0	1	yes
C	1	1	1	1	no
B	1	1	0	1	no
D	0	0	1	0	no
C	0	0	0	1	no
A	1	1	1	1	no
D	0	1	0	1	yes
C	1	0	1	1	no
C	0	0	0	1	no
C	1	0	0	1	yes
B	1	1	1	1	no
B	0	0	0	1	yes
D	0	1	0	1	no
C	0	0	0	1	yes
D	1	1	0	1	no
C	0	0	0	1	no
D	1	0	0	1	yes
C	1	0	1	1	yes
B	0	0	0	1	no
B	1	1	0	1	no
B	0	0	0	1	no
B	0	0	1	1	no
C	1	1	0	1	yes
B	0	0	0	1	no
C	1	0	1	1	no

B	1	1	0	1	no
C	1	1	0	1	yes
D	1	0	0	1	no
C	0	0	1	1	no
B	0	0	0	1	yes
C	1	1	1	1	no
D	1	0	0	0	no
C	1	0	0	0	no
B	0	1	0	0	no
D	0	1	0	0	no
B	1	0	0	0	no
C	0	1	0	0	no
D	1	0	0	0	yes
C	0	1	0	0	yes
A	0	1	0	0	no
C	1	0	0	0	no
A	0	1	0	0	no
C	0	1	0	0	no
A	1	0	0	0	no
B	0	1	0	0	no
A	1	0	0	0	no
A	1	0	0	0	no
A	1	0	0	0	no
B	0	1	1	0	no
A	1	0	1	0	no
A	1	0	0	0	no
A	0	1	1	1	no
B	1	1	1	1	yes
A	1	1	1	0	no
B	1	1	0	0	no
B	0	1	1	1	no
B	1	1	1	1	no
D	1	0	0	0	no
C	1	1	1	1	yes
C	1	1	1	1	no
B	0	1	1	0	no
B	0	0	0	1	no
B	1	1	1	1	no
B	1	1	1	1	no
A	1	1	1	0	no
A	1	0	0	0	no
A	0	1	1	1	no
C	0	1	1	0	no
D	0	0	1	0	yes
C	1	0	0	1	no
A	0	1	1	1	no
A	1	1	1	1	no

B	1	1	1	0	no
D	1	1	0	0	no
A	1	1	0	1	yes
B	1	1	1	1	yes
D	0	0	1	0	no
B	1	1	1	1	no
D	1	0	1	1	no
C	1	1	0	0	no
A	0	0	0	1	no
B	1	1	1	1	no
B	1	1	1	1	no
C	0	0	0	1	no
D	1	1	0	1	yes
D	0	0	1	0	no
C	1	1	0	0	no
C	1	1	1	1	yes
B	1	0	0	1	yes
B	1	0	1	0	no
